# Immunity-Related Gene Signature Identifies Subtypes Benefitting From Adjuvant Chemotherapy or Potentially Responding to PD1/PD-L1 Blockage in Pancreatic Cancer

**DOI:** 10.3389/fcell.2021.682261

**Published:** 2021-06-23

**Authors:** Hao Qian, Hongzhe Li, Junjie Xie, Xiongxiong Lu, Fanlu Li, Weishen Wang, Xiaomei Tang, Minmin Shi, Linxi Jiang, Hongwei Li, Hao Chen, Chenghong Peng, Zhiwei Xu, Xiaxing Deng, Baiyong Shen

**Affiliations:** ^1^Department of General Surgery, Pancreatic Disease Center, Ruijin Hospital, Shanghai Jiao Tong University School of Medicine, Shanghai, China; ^2^Research Institute of Pancreatic Diseases, Shanghai Jiao Tong University School of Medicine, Shanghai, China; ^3^State Key Laboratory of Oncogenes and Related Genes, Shanghai, China; ^4^Institute of Translational Medicine, Shanghai Jiao Tong University, Shanghai, China

**Keywords:** gene signature, immunity, pancreatic cancer, pancreatic ductal adenocarcinoma, prognosis

## Abstract

Tumor microenvironment comprises of a variety of cell types, which is quite complex and involved in chemotherapy and immune checkpoint blockage resistance. In order to explore the mechanisms involved in tumor immune microenvironment in pancreatic ductal adenocarcinoma (PDAC), we first constructed an immunity-related 18-gene signature using The Cancer Genome Atlas (TCGA) PDAC project data. Then we applied the 18-gene signature to divide PDAC patients into low score and high score groups. Patients in high score group showed inferior prognosis, which was validated in another four independent cohorts, including Ruijin cohort. High score group showed significant enrichment of pathways involved in cell division and cell cycle especially in G1/S phase transition. In high score group, IHC analysis revealed higher levels of the proliferative indexes of Ki67 and PCNA than that in low score group. Prognostic analysis confirmed that patients in high score group could benefit from the gemcitabine-based adjuvant chemotherapy. In low score group, the programmed cell death 1 ligand 1(PD-L1) (+) cases showed worse prognosis but higher T cell infiltration than PD-L1(−) cases. Our immunity-related 18-gene signature could effectively predict PDAC prognosis, and it might be a practical predictive tool to identify PDAC subtype benefitting from gemcitabine-based adjuvant chemotherapy or potentially responding to PD1/PD-L1 blockade therapy.

## Introduction

Pancreatic cancer is a fatal malignancy with extremely poor prognosis (Sodir et al., 2020), accounting for an estimated 57,600 new cases and 47,050 deaths annually (Siegel et al., 2020). Owing to its anatomical and pathological features, pancreatic cancer occurs occultly and grows rapidly. Most patients have lost their chance of surgery when diagnosed (Strobel et al., 2019). Although significant improvement has been achieved in pancreatic cancer treatment, the 5-year survival rate of pancreatic ductal adenocarcinoma (PDAC) is still rather low (Zhu et al., 2018).

Immunity is the essential component of the tumor microenvironment, which plays a pivotal role in tumor initiation, progression, and metastasis (Qian and Pollard, 2010; Berraondo et al., 2016; Keren et al., 2018). Each immune subtype harbors different functions and can be used to predict the states of tumors (Mollaoglu et al., 2018). In the previous study, researchers showed that interfering with immune conditions by targeting specific molecules could suppress tumor progression and improve the effectiveness of chemotherapy (Galluzzi et al., 2015). Among the specific molecules, those mainly expressed on the cell membrane could maintain self-tolerance and modulate immune responses by triggering immunosuppressive signaling pathways, defining them as immune checkpoints (Wykes and Lewin, 2018). Novel therapy targeting immune checkpoints, such as programmed cell death 1 (PD1)/programmed cell death 1 ligand 1 (PD-L1), cytotoxic T-lymphocyte associated protein 4 (CTLA4) and T-cell membrane protein 3 (TIM3) have made breakthrough in many types of cancer treatment (Sharma and Allison, 2015; Topalian et al., 2016). However, only a fraction of patients could acquire significant effect from immune checkpoint blockade (Zappasodi et al., 2018). PDAC patients responding little to immune checkpoints blockade may be due to the insensitive immune microenvironment (Zhao et al., 2019). In a phase II trial, the advanced PDACs could not benefit from anti-CTLA4 (Ipilimumab) treatment (Royal et al., 2010). In addition, anti-PD-L1 immunotherapy also showed limited effects in PDAC in a phase I trial (Brahmer et al., 2012). Many studies have revealed some factors involved in the sensitivity of immune checkpoint blockade, such as the expression of targeted molecules, microsatellite instability, mutation load, and immune infiltration (Catalano et al., 2019; Mandal et al., 2019). For example, the tumors with CD3(+) and CD8(+) T cell infiltration were sensitive to anti-PD-L1 and anti-CTLA4 immunotherapy (Foy et al., 2017; Wu et al., 2018). Thus, exploring the tumor immune microenvironment could help us make the personalized treatment of immune checkpoint blockade in PDAC.

The method of combining immunity-related genes with clinical characteristics has been applied to predict prognosis, recurrence and response to therapy in multiple cancers (Wu et al., 2018). In a previous study, researchers constructed a stromal immunotype to predict patients’ overall survival (OS) and diseases free survival (DFS) in bladder cancer (Foy et al., 2017). In gastric cancer, a least absolute shrinkage and selection operator (LASSO) Cox regression model was established to predict patients’ prognosis and identify the subgroup suitable for adjuvant chemotherapy (Cheadle et al., 2003). These studies revealed the detailed mechanisms of the tumor immune microenvironment and provided significant indications for clinical therapy.

In the present study, we aimed to develop an immunity-related gene signature based on LASSO Cox regression to predict patients’ outcomes using data from TCGA, which was further validated in another four independent cohorts from the International Cancer Genome Consortium (ICGC), the Gene Expression Omnibus (GEO), and Ruijin cohort. Furthermore, the signature could be used to identify PDAC subtype benefitting from gemcitabine-based adjuvant chemotherapy or possibly responding to anti-PD1/PD-L1 immunotherapy.

## Materials and Methods

### PDAC Datasets Extraction and Data Processing

Raw count data and corresponding clinical characteristics of 173 patients with PDAC were downloaded from the TCGA database^1^. ICGC CA (Canada) raw RNA sequencing dataset and corresponding clinical characteristics of 115 patients, and the ICGC AU (Australia) gene expression microarray dataset and corresponding clinical characteristics of 68 patients were downloaded from the ICGC database^2^. The GEO dataset GSE57495 and corresponding clinical characteristics of 63 patients were downloaded from GEO database^3^. RNA-sequencing data was normalized by transcript per million (TPM), and gene expression was calculated as log2 (TPM + 1). For the gene expression microarray, if one gene was detected using multiple probes, the probe with the maximum average used. Then, gene expression values were normalized by log2 transformation. To remove the batch effects of different platform in this study, the expression values of each gene were z-score transformed.

### Identification of Immunity-Associated Genes

By interrogating the ImmPort database^4^, we obtained a total of 1,811 immunity-related genes. After matching with genes in the TCGA database, 1,308 immunity-related genes were used for further analysis.

### Patients

A total of 101 fresh frozen primary PDAC samples were utilized as the validation cohort (Ruijin cohort), which were collected consecutively at Ruijin Hospital from April 2012 to November 2014. The inclusion and exclusion criteria were as follows: (1) Pathologically diagnosed as having pancreatic ductal adenocarcinoma (PDAC) without any other types of pancreatic cancer; (2) without other malignant cancers; and (3) did not receive any preoperative adjuvant therapy. The clinicopathological variables are listed in Supplementary Table 2. All patients provide signed informed consent. The study was approved by the Ethical Committee of Ruijin Hospital, Shanghai Jiao Tong University School of Medicine, Shanghai, China.

### RNA Extraction and Quantitative Real-Time Reverse Transcription PCR (qRT-PCR)

Total RNA from 101 PDAC samples (Ruijin cohort) was extracted using the TRIzol reagent (Invitrogen, Waltham, MA, United States) according to the manufacture’s protocol. Reverse-transcription PCR was performed using a Reverse Transcription kit (TOYOBO, Osaka, Japan). Quantitative real-time PCR was carried out in 10 μl reaction mixtures with an HT 7900 machine (Applied Biosystems, Foster City, CA, United States) using SYBR^TM^ Select Master Mix (Applied Biosystem). The gene primers were designed and synthesized by Sangon Biotech (Shanghai, China), and are listed in Supplementary Table 3. *GAPDH* (encoding glyceraldehyde-3-phosphate dehydrogenase) was applied as an internal control. Gene expression was normalized as −ΔCT = − (CT gene – CT GAPDH). Finally, expression values of each gene were z-score transformed (Cheadle et al., 2003).

### Identification and Validation of the Immunity-Related Gene Signature

First, we used the TCGA PDAC dataset as the training cohort. Using univariate Cox analysis, immunity-related genes that were significantly associated with good or poor prognosis were identified using the “Survival” package in the R software^5^. The prognostic genes were displayed by a forest plot using “forestplot” in R. Then, LASSO Cox regression was performed to generate a prognostic signature with the immunity-related genes using the “glmnet” package in R (Liu et al., 2019). Finally, a nomogram based on 18 immunity-related genes was plotted using the “rms” package in R, and the corresponding formula was extracted using the “nomogramEx” package in R. According the risk score and survival status of every patient, optimal cutoff values were set and all patients could be assigned to a high score or low score group.

### Gene Ontology (GO) and Kyoto Encyclopedia of Genes and Genomes (KEGG) Pathway Analyses

By comparing the differentially expressed genes (DEGs) between the high score or low score group using the “limma” package in R, we obtained the significantly changed genes between the two groups. The gene names were imported into the Metascape database, and GO and KEGG pathway analyses were performed. The significantly enriched pathways were displayed in a histogram (*p* < 0.01).

### Gene Set Variation Analysis (GSVA) and Gene Set Enrichment Analysis (GSEA)

The GSVA analysis was performed using “GSVA” package in R. The gene sets using in GSVA analysis were downloaded from GSEA molecular database^6^. T cell immunoreaction and PD1-related immunosuppressive pathways were extracted and used for GSVA analysis. As for GSEA analysis (Mootha et al., 2003; Subramanian et al., 2005), we construct a gemcitabine resistance related gene set by comparing the DEGs between the gemcitabine resistance population and main tumor cell population in GSE 36563 dataset and using genes with *p* value < 0.05 and | Log2 fold change | > 1. The gemcitabine resistance related gene set was shown in Supplementary Table 4.

### Immunohistochemistry (IHC)

Of the above 101 PDAC samples, 81 tissue specimens were fixed using 10% neutral buffered formaldehyde, and then embedded in paraffin. Tissue sections were cut into 5-μm thick slices, which were coated with 3-aminopropyltriethoxysilane. Expression of CD3 + T cell, CD8 + T cell, and PD-L1 in these paraffin-embedded tissue sections were examined by IHC using the streptavidin-peroxidase method. A rabbit anti-human Ki67 monoclonal antibody (dilution, 1:250; catalog no. ab16667; Abcam, Cambridge, United Kingdom), a rabbit anti-human PCNA monoclonal antibody (dilution, 1:250; catalog no. ab265609; Abcam, Cambridge, United Kingdom), a rabbit anti-human PD-L1 monoclonal antibody (dilution, 1:250; catalog no. ab 213524; Abcam, Cambridge, United Kingdom), a rabbit anti-human CD3 monoclonal antibody (dilution, 1:150; catalog no. ab135372; Abcam), a rabbit anti-human CD8 monoclonal antibody (dilution, 1:250; catalog no. ab93278; Abcam) were used. The experimental procedure was as follows: (1) Slides were baked at 65°C for 2 h, deparaffinized in xylene four times (8 min each time), and then rehydrated in 100, 95, 85, and 75% ethanol successively (5 min each time). For antigen retrieval, the sections were autoclaved at 121°C for 10 min in citrate buffer (10 mmol/l sodium citrate; pH 6.0). By incubating the slides in 0.3% H_2_O_2_ solution, the endogenous peroxidase activity was blocked. After blocking with normal goat serum, the sections were incubated with the primary antibodies overnight at 4°C. Secondary antibodies (goat anti-rabbit antibody; 1:100 dilution; cat no. CW2069A; CWBio, Beijing, China) was incubated with the tissue sections for 15 min at room temperature. Finally, the slides were stained with 3, 3-diaminobenzidine tetrahydrochloride (DAB) and the nuclei were counterstained with hematoxylin. To semi-quantify the expression of Ki67, PCNA, and PD-L1 in PDAC tissue, we referenced both the proportion and intensity of stained tumor cells. Proportion scores: <5%, 5–25%, 25–50%, 50–75%, and ≥75% were recorded as 0, 1, 2, 3, 4, respectively. Staining scores: negative, weak, moderate, and strong staining were recorded as 0, 1, 2, 3, respectively. Finally, IHC scores was calculated as “proportion score × intensity score.” For each case, five high power fields (400×) were evaluated and averages were calculated.

### Statistical Analysis

All statistical analyses were performed using GraphPad Prism 7.0 (GraphPad Software Inc., La Jolla, CA, United States). The Kaplan-Meier method was used to plot survival curves, and the log-rank test was used to assess intergroup differences. Difference between two groups was assessed using Student’s *t*-test. The χ^2^ test was carried out to analyze the relationship between the 18-gene signature and clinical characteristics. *P* < 0.05 were considered statistically significant.

## Results

### Characteristics of Patients With PDAC

**FIGURE 1:**
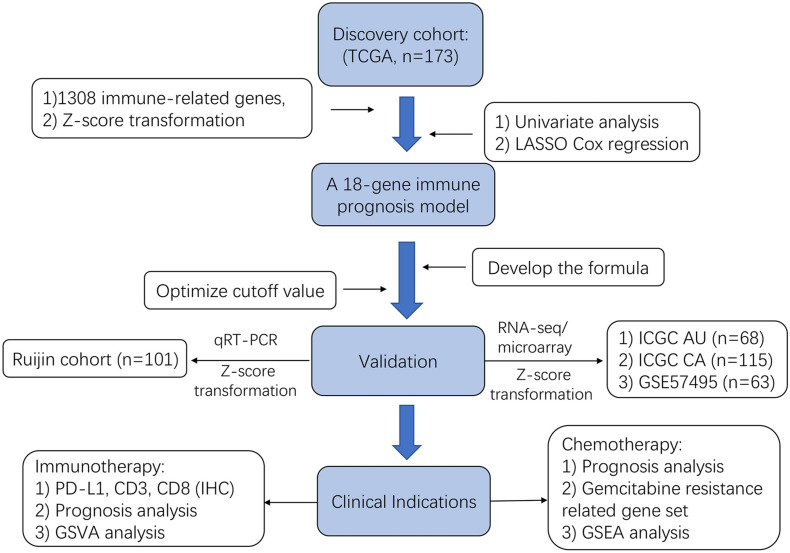
Flowchart presenting the process of construction of immunity-related 18-gene signature, validation and clinical significance relevance in this study.

The schematic flow chart of this study is shown in Figure 1. Our study totally enrolled 520 patients diagnosed with PDAC. Among them, the TCGA (*n* = 173) patients with PDAC were assigned as the training cohort, and the ICGC AU (*n* = 68), ICGC CA (*n* = 115) and GSE57495 (*n* = 63) PDAC patients were assigned as validation cohorts. Furthermore, patients (*n* = 101) with PDAC from Ruijin hospital, Shanghai Jiao Tong University School of Medicine were used as another independent validation cohort. The characteristics of the patients from the TCGA database and Ruijin cohort are shown in Tables 1, 2, respectively.

**TABLE 1:** The association of the immune signature with clinicopathological characteristics in TCGA database.

Features	Total	Score level		*p-value*
		High	Low	
**Gender**				0.4997
**Male**	94	63	31	
**Female**	78	56	22	
**Age**				0.1493
**≤60**	58	36	22	
**>60**	114	83	31	
**T**				0.0078*
**T1 + T2**	30	15	15	
**T3 + T4**	141	105	36	
**N**				0.0121*
**N0**	48	27	21	
**N1**	120	91	29	
**M**				0.5745
**M0**	76	55	21	
**M1**	4	2	2	
**TNM stage**				0.0018*
**I**	20	8	12	
**II + III + IV**	150	111	39	
**Histologic Grade**				0.0469*
**G1**	29	14	15	
**G2**	93	67	26	
**G3**	48	36	12	
**G4**	2	2	0	

*P < 0.05.

**TABLE 2:** The association of the immune signature with clinicopathological characteristics in Ruijin cohort.

Features	Total	Score level		*p-value*
		High	Low	
**Gender**				0.2518
**Male**	63	42	21	
**Female**	38	21	17	
**Age**				0.0757
**≤60**	46	33	13	
**>60**	55	30	25	
**Tumor size**				0.5461
**≤4 cm**	82	50	32	
**>4 cm**	19	13	6	
**Vascular invasion**				0.7750
**Yes**	55	35	20	
**No**	46	28	18	
**Lymph node metastasis**				0.3263
**Yes**	62	41	21	
**No**	39	22	17	
**Differentiation**				0.0145*
**Well/moderate**		15	18	
**Poor**		48	20	
**TNM stage**				0.1340
**I**	19	9	10	
**II + III + IV**	82	54	28	

*P < 0.05.

### Identification and Validation of Immunity-Associated Gene Signature in PDAC

**FIGURE 2:**
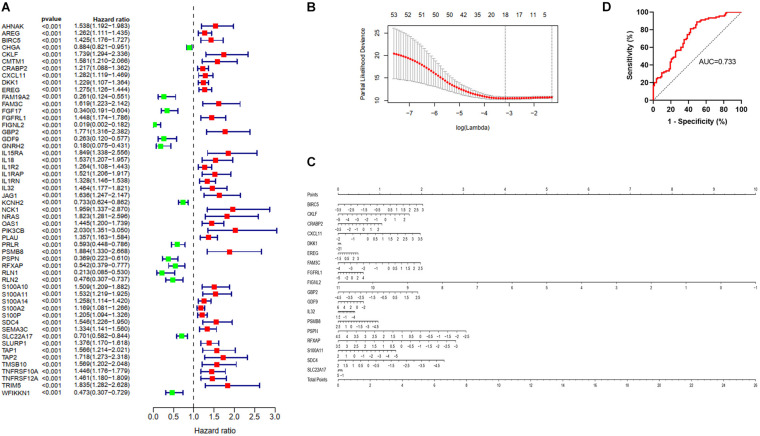
Identification and validation of the immune predictive model. **(A)** A forest plot showing associations between the 53 proteins whose *p* value were less than 0.001 and overall survival in the training group. Unadjusted hazard ratios are shown with 95% confidence intervals. **(B)** The cross-test for selecting parameters in the LASSO model. Lambda represents the selected parameters, and partial likelihood deviance is plotted against log (lambda). **(C)** Nomogram predicting the probability overall survival. Eighteen genes were used to assign the points and draw a line depending on the corresponding values. “Total points,” The sum of these 18 genes’ points makes up the “Total points” and can predict overall survival. **(D)** The ROC curve of the TCGA (*n* = 173) training model for predicting patients’ overall survival.

**FIGURE 3:**
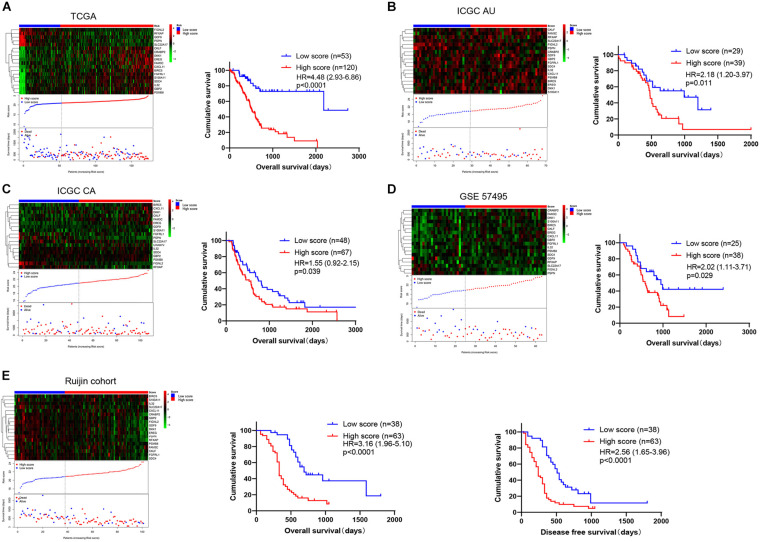
The distribution and Kaplan-Meier survival curves of the immunity-related 18-gene signature depends on the patients’ risk score. The risk score for all patients with PDAC were plotted in ascending order and marked as low score (blue) or high score (red). The survival status of the patients is marked as dead (red) and alive (blue). The proportion of patients who died in the high score group is obviously higher compared with that in the low score group. **(A)** TCGA (*n* = 173), **(B)** ICGC AU (*n* = 68), **(C)** ICGC CA (*n* = 115), **(D)** GSE57495 (*n* = 63), and **(E)** Ruijin cohort (*n* = 101) are shown, respectively, and the Ruijin cohort also exhibited the DFS.

We matched immunity-related genes from the ImmPort database with genes in the TCGA database and 1308 immunity-related genes (Supplementary Table 1) were obtained for further analysis. By performing univariate Cox regression analysis, 53 genes whose *p* values were less than 0.001 were chosen as candidates (Figure 2A). We then used the LASSO Cox regression algorithm and a total of 18 genes were identified to develop a risk score classifier (Figure 2B). A nomogram of the 18 genes predicting the probability of overall survival is shown in Figure 2C. The formula of risk score calculation was illustrated in Supplementary Table 2. We used a receiver operating characteristic (ROC) curve (Figure 2D) to test the effectiveness and determine the best cutoff value of the risk scores. The area under the curve (AUC) was 0.733, and 20.91 was identified as the optimal cutoff value. We divided patients into high score and low score group using the 18 immunity-related classifier and plotted the Kaplan-Meier survival curves. We found that patients in the low score group had more favorable prognosis than patients in the high score group, both in training and validation cohorts. In TCGA database (Figure 3A), patients in the low score group had significantly longer OS than patients in the high score group (HR = 4.48 (2.93–6.86), *p* < 0.0001). In the other four validation cohorts (Figures 3B–E), patients in the high score group also had inferior outcomes compared with those in the low score group (ICGC AU cohort: HR = 2.18 (1.20–3.97), *p* = 0.011; ICGC CA cohort: HR = 1.55 (0.92–2.15), *p* = 0.039; GSE57495 cohort: HR = 2.02 (1.11–3.71), *p* = 0.029; Ruijin cohort: HR = 3.16 (1.96–5.10), *p* < 0.0001). In Ruijin cohort, the phenomenon also applied to DFS, with an HR of 2.56 (1.65–3.96, *p* < 0.0001). We also found that the immune signature was significantly related to TNM stage and histological grade in the TCGA database (Table 1), and similar results were observed in Ruijin cohort (Table 2).

### Prognosis Prediction of Immunity-Related Gene Signature Is Superior to TMN Stage

After constructing the immunity-related 18-gene classifier, we performed univariate and multivariate Cox regression analysis involved in our immune signature and multiple clinicopathological features, such as TNM stage (II + III + IV vs. I), the CA19-9 level (>200 kU/L vs. ≤200 kU/L), the adjuvant chemotherapy status (yes or no), and PD-L1 expression. The CA 19-9 level was a moderate risk predictor in PDAC and we assigned 200 kU/L as boundary according to previous studies (Ballehaninna and Chamberlain, 2012; Aziz et al., 2019). In TCGA cohort, our immune signature (*p* < 0.001, HR = 5.09), TNM stage (*p* = 0.026, HR = 2.58), differentiation (*p* = 0.042, HR = 1.57), chemotherapy status (*p* = 0.021, HR = 0.61) and PD-L1 (*p* = 0.035, HR = 1.38) were found to be effective predictors of OS by univariate Cox regression analysis (Supplementary Figure 1A). Then we took these factors together to perform multivariate Cox regression analysis. Interestingly, our immune signature (*p* < 0.001, HR = 4.86) and chemotherapy status (*p* < 0.001, HR = 0.40) were independent prognosis factors (Supplementary Figure 1B). In Ruijin cohort, our immune signature (*p* < 0.001, HR = 3.65), CA 19-9 level (*p* = 0.003, HR = 2.05) and differentiation (*p* = 0.002, HR = 2.42) were significantly related to OS by univariate Cox regression analysis (Supplementary Figure 1C). Multivariate analysis revealed that our immune signature (*p* < 0.001, HR = 3.45), CA 19-9 level (*p* = 0.002, HR = 2.18) and differentiation (*p* = 0.012, HR = 2.05) could serve as independent predictors for OS in PDAC (Supplementary Figure 1D).

**FIGURE 4:**
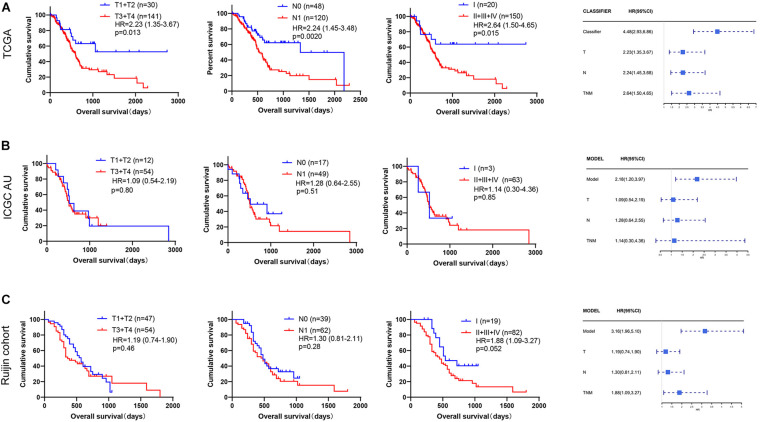
Comparison of prognostic prediction ability between our immune signature and TNM stage. The Kaplan-Meier survival curves according to TNM stage, and forest plots show the HR value of our immune signature and TNM stage in **(A)** TCGA (*n* = 173), **(B)** ICGC AU (*n* = 68), and **(C)** Ruijin cohort (*n* = 101).

In order to compare the prognosis prediction ability between the immune signature and T stage, N stage, and TNM stage, we also plotted the Kaplan-Meier survival curves of these clinicopathological features, and the *p* value and HRs were obtained by log rank test. In the TCGA data (Figures 3A, 4A), the HR value of our immune signature (HR = 4.48, 2.93–6.86) was superior to T stage (HR = 2.23, 1.35–3.67), N stage (HR = 2.24, 1.45–3.68) and TNM stage (HR = 2.64, 1.50–4.65). Similar results were observed in the ICGC AU and Ruijin cohorts (Figures 3B,E, 4B,C).

### The Immunity-Related 18-Gene Signature Predicts Patients’ Response to Adjuvant Chemotherapy in PDAC

**FIGURE 5:**
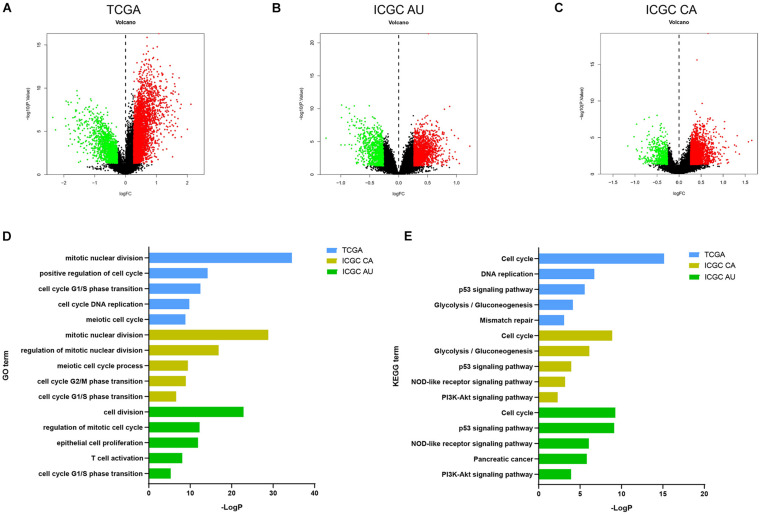
Pathways involved in the immunity-related 18-gene signature. The volcano plot of DEGs in **(A)** TCGA, **(B)** ICGC AU, **(C)** ICGC CA databases. The volcano plots were constructed using fold-change and *p* values. Genes with fold-change > 1.2 and *p* < 0.05 were used to pathways analysis. **(D,E)** Similar GO and KEGG pathways enriched in high score group in TCGA, ICGC CA, and ICGC AU.

**FIGURE 6:**
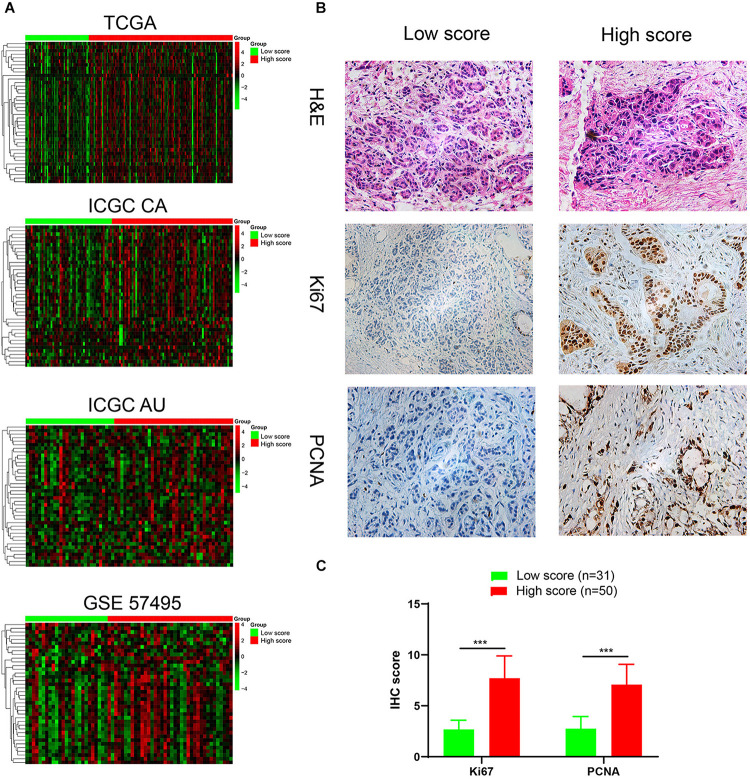
Identification and validation of a highly proliferative subgroup of PDAC. **(A)** Heatmap showed that G1/S transition pathway was obviously enriched in high score group. We used kmeans clustering to draw heatmaps, and the lines left to heatmaps represent that the genes at ends of the lines had a high correlation. And the gene names in each row from top to bottom are ANXA1, AURKA, BCAT1, C10orf99, CCNA2, CCNB1, CCND1, CCNE1, CDC25C, CDC45, CDC6, CDK1, CDK2, CDK2AP2, CDK6, CDKN3, CDT1, E2F1, E2F7, E2F8, EGFR, EIF4EBP1, EZH2, GMNN, GTSE1, IQGAP3, KIF14, MCM10, MCM2, MCM4, MUC1, ORC1, ORC6, PML, PSME2, RCC1, RRM2, SFN, TNKS1BP1, TYMS. **(B)** H&E staining and immunohistochemical analyses of Ki-67, PCNA were performed on the tumor sections (low score group, *n* = 31; high score group, *n* = 50) **(C)** The IHC scores of Ki-67 and PCNA in low score (*n* = 31) and high score (*n* = 50) groups. ***P < 0.001.

To interrogate potential signaling pathways involving in our immune signature in PDAC, we compared differentially expressed genes between high score group and low score group, and selected the genes with fold-change > 1.2 and p.adjust value < 0.05 to perform the GO and KEGG enrichment analyses (Figures 5A–C and Supplementary Figures 2A–C). Similar enriched pathways from the 3 datasets were displayed in Figures 5D,E. Cell cycle, cell division, p53 signaling pathways were significantly enriched in high score group, which indicated that it harbored higher proliferative potential. It is well known that gemcitabine exerts antitumor activity mainly by targeting G1/S phase. In GO term of G1/S phase transition, 40 genes were significantly enriched in high score group in TCGA cohort, and similar results were observed in another three independent cohorts (Figure 6A). Then H&E staining was used to detect the proliferation related markers (Ki67 and PCNA) in 81 PDAC patients (low score group, *n* = 31; high score group, *n* = 50) in Ruijin cohort. The IHC analysis verified that higher levels of Ki67 and PCNA in high score group than that in low score group (Figures 6B,C). These results indicated that patients in high score group might be more responsive to chemotherapy targeting cell cycle (Venkatasubbarao et al., 2013).

**FIGURE 7:**
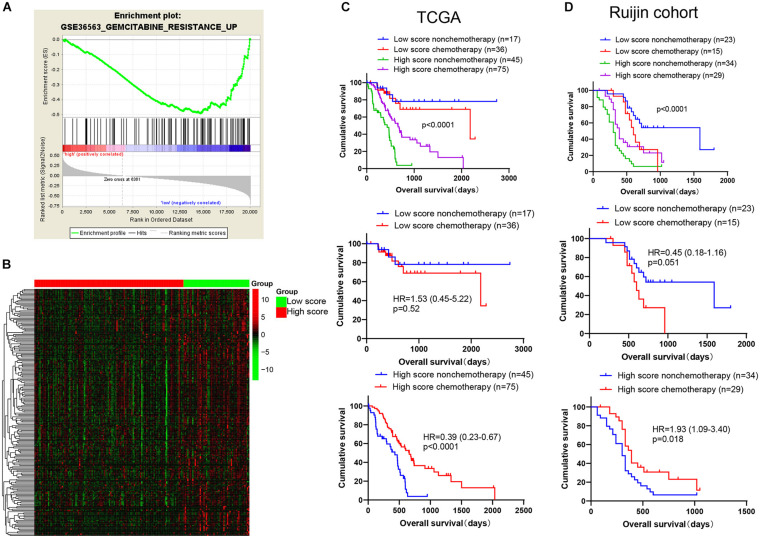
Benefit of gemcitabine-based chemotherapy among patients in high and low score groups. **(A)** The gemcitabine resistance related gene set was significantly enriched in the low score group of TCGA cohort by GSEA analysis. The green curve represents enrichment score (ES). The highest point was used to represent ES value in gemcitabine resistance pathway. The ES value indicates the correlation between gene and gemcitabine resistance pathway. The black bar codes represent genes in gemcitabine resistance pathway and these genes were ordered according to their expression levels. The left end or right end genes are leading edge subset strongly contributed to ES value. The bottom numbers represent the order of expression levels in the genome from highest to lowest. **(B)** The 198 gemcitabine resistance-related genes were significantly attenuated in high score group of TCGA cohort (*p* < 0.05). Patients from the **(C)** TCGA database (*n* = 173) and **(D)** Ruijin cohort (*n* = 101) are divided into four groups (TCGA, Low score non-chemotherapy, *n* = 17, Low score chemotherapy, *n* = 36, high score non-chemotherapy, *n* = 45, high score chemotherapy, *n* = 75; Ruijin, Low score non-chemotherapy, *n* = 23, Low score chemotherapy, *n* = 15, high score non-chemotherapy, *n* = 34, high score chemotherapy, *n* = 29) and then Kaplan-Meier survival curves were plotted.

As the first-line medicine for chemotherapy, gemcitabine has proven its effectiveness in PDAC (Fuchs et al., 2015). Cells possessing a vigorous proliferation ability are more sensitive to gemcitabine therapy (Zheng et al., 2015). Thus, we suspected that patients might be more sensitive to gemcitabine-based chemotherapy in high score group. We further constructed a gemcitabine resistance related gene set by using GSE36563 dataset (Van den Broeck et al., 2012), which contained 484 genes upregulated in gemcitabine resistance group, named as GEMCITABINE_RESISTANCE_UP. GSEA analysis showed that the gene set was significantly enriched in low score group in TCGA cohort (Figure 7A), and heatmap was further plotted to display 198 significantly attenuated genes of this gene set in high score group (Figure 7B and Supplementary Table 5). Prognosis analysis showed that the patients in high score group could benefit from chemotherapy in both the TCGA and Ruijin cohorts [Figures 7C,D, TCGA: chemotherapy vs. non-chemotherapy, *p* < 0.0001, HR = 0.39(0.23–0.67); Ruijin: chemotherapy vs. non-chemotherapy, *p* = 0.0083, HR = 0.46(0.25–0.82)]. However, the patients in low score group did not display this phenomenon [Figure 7C, TCGA: chemotherapy vs. non-chemotherapy, *p* = 0.52, HR = 1.53(0.45–5.22); Ruijin: chemotherapy vs. non-chemotherapy, *p* = 0.21, HR = 1.72(0.69–4.26)].

### Identification of a Lymphocyte-Infiltrated PD-L1(+) PDAC Subgroup Associated With Poor Prognosis

**FIGURE 8:**
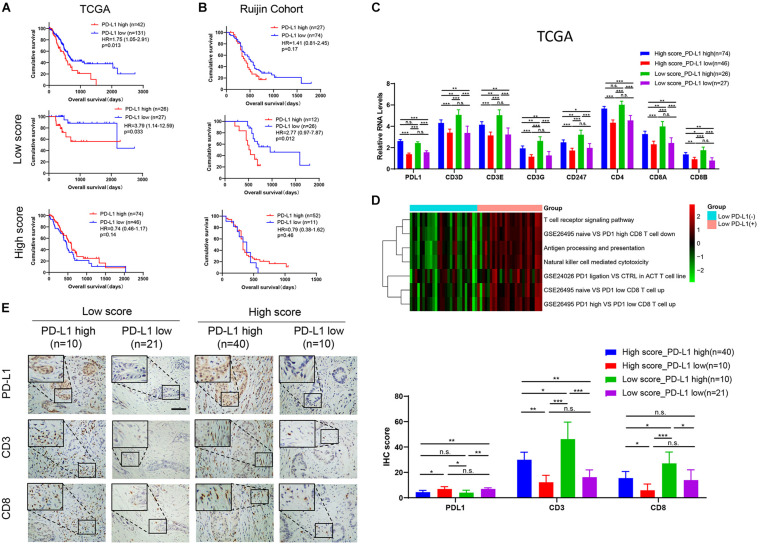
PD-L1(+) cases in low score group show inferior prognosis and lymphocytes infiltration. Kaplan-Meier survival curves of PD-L1 expression in all patients, the low score group, and the high score group in **(A)** TCGA (High score: PD-L1 high, *n* = 74; PD-L1 low, *n* = 46. Low score: PD-L1 high, *n* = 26; PD-L1 low, *n* = 27) and **(B)** Ruijin cohort (High score: PD-L1 high, *n* = 52; PD-L1 low, *n* = 11. Low score: PD-L1 high, *n* = 12; PD-L1 low, *n* = 26). **(C)** The relative RNA level of immune infiltration markers in the TCGA database, including PD-L1, CD3D, CD3E, CD3G, CD247, CD4, CD8A, CD8B. **(D)** Heatmap showed that higher GSVA scores of T cell immunoreaction pathways and PD1-related immunosuppressive pathways in PD-L1(+) than in PD-L1(–) cases in low score group. **(E)** IHC profile and corresponding scores of PD-L1, CD3 and CD8 in four groups (High score_PD-L1 high, *n* = 40; High score_PD-L1 low, *n* = 10; Low score_PD-L1 high, *n* = 10; Low score_PD-L1 high, *n* = 21). *p < 0.05; **p < 0.01; ***p < 0.001; n.s., *p* > 0.05.

The immunotherapy targeting PD-L1 showed impressive anti-tumor activity. In this study, PD-L1 expression could successfully predict the patients’ outcome in PDAC in the TCGA data (Figure 8A), but not in the other four interdependent cohorts (Figure 8B, Supplementary Figures 3A–C). However, we found that low expression of PD-L1 was significantly related to longer OS in low score group in TCGA and Ruijin cohorts. In TCGA cohort (Figure 8A), patients with high PD-L1 expression had poorer OS than patients with low PD-L1 expression in low score group, with an HR of 3.79 (1.14–12.59, *p* = 0.033). In the Ruijin cohort (Figure 8B), patients with low PD-L1 expression had favorable outcomes compared with patients with high PD-L1in low score group, with an HR against low PD-L1 expression 1of 2.93 (1.01–8.45, *p* = 0.0092). Although the results did not show significant differences, we discovered the same trend in low score group of the ICGC AU cohort and GSE 57495 cohort (Supplementary Figures 3B,C). However, in high score group, PD-L1 expression showed the reverse function in the ICGC CA and ICGC AU cohorts [ICGC CA: PD-L1 high vs. PD-L1 low, *p* = 0.035, HR = 0.58(0.32–1.03); ICGC AU: PD-L1 high vs. PD-L1 low, *p* = 0.032, HR = 0.37(0.09–1.52)]. The mRNA level of PD-L1 expression did not show significant differences between low score and high score groups (Supplementary Figure 3D). In TCGA database, the CD3D, CD3E, CD3G, CD247, CD4, CD8A, and CD8B RNA levels were the highest in Low score_PD-L1 high group (Figure 8C). Moreover, we observed not only the activation of T cell immunoreaction pathways in low score group, but also the activation of PD1-related immunosuppressive pathways by GSVA analysis (Figure 8D). In the Ruijin cohort, the IHC result showed higher numbers of CD3(+) T cells and CD8(+) T cells in PD-L1(+) cases in low score group (Figure 8E, all *p* < 0.05). The above results indicated that PD-L1(+) cases in low score group displayed stronger immune infiltration and may be suitable for PD1/PD-L1 blockade immunotherapy.

**FIGURE 9:**
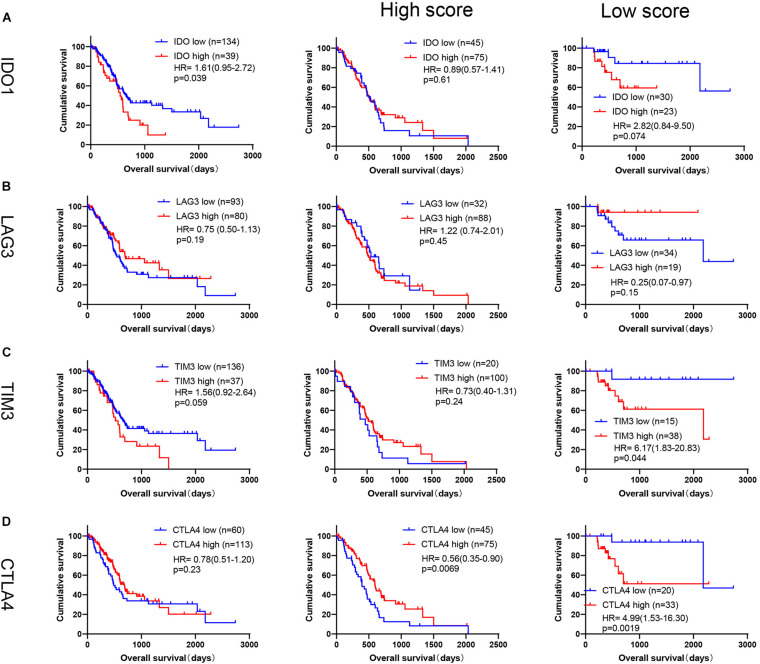
Prognosis analysis of TIM3, CTLA4, IDO1 and LAG3 in TCGA database. Kaplan-Meier survival curves of **(A)** IDO1 (High score: PD-L1 high, *n* = 75; PD-L1 low, *n* = 45. Low score: PD-L1 high, *n* = 23; PD-L1 low, *n* = 30), **(B)** LAG3 (High score: PD-L1 high, *n* = 88; PD-L1 low, *n* = 32. Low score: PD-L1 high, *n* = 19; PD-L1 low, *n* = 34), **(C)** TIM3 (High score: PD-L1 high, *n* = 100; PD-L1 low, *n* = 20. Low score: PD-L1 high, *n* = 38; PD-L1 low, *n* = 15), **(D)** CTLA4 (High score: PD-L1 high, *n* = 75; PD-L1 low, *n* = 45. Low score: PD-L1 high, *n* = 33; PD-L1 low, *n* = 20) expression in all patients, or by subgroup analysis based on our immune signature in TCGA database.

We also performed prognosis of other immune checkpoints in PDAC, such as IDO1, LAG3, TIM3, and CTLA4. As shown in Figures 9A–D, high expression of IDO1 was associated with poor outcome in PDAC in TCGA database [IDO1: *p* = 0.039, HR = 1.61(0.95–2.72)], but no significant difference for LAG3 [*p* = 0.19, HR = 0.75(0.50–1.13)], TIM3 [*p* = 0.059, HR = 1.56(0.92–2.64)] and CTLA4 [*p* = 0.23, HR = 0.78(0.51–1.20)]. However, by conducting subgroup analysis based on our immune signature, high levels of TIM3 and CTLA4 were significantly associated with poor OS in low score group [Figures 9A–D, TIM3: *p* = 0.044, 6.17 (1.83–20.83); CTLA4: *p* = 0.0019, HR = 4.99(1.53–16.30)], but not for LAG3 [Figure 9B, *p* = 0.15, HR = 0.25(0.07–0.97)]. High level of IDO1 also tended to be related to short OS in low score group [Figure 9A, IDO1: *p* = 0.074, HR = 2.82(0.84–9.50)], but did not show significant difference, which may be due to small sample size (*n* = 53). Because TIM3 and CTLA4 expression could significantly predict OS in low score group in TCGA database, we further performed the above analyses of TIM3 and CTLA4 in ICGC CA, ICGC AU and GSE57495 databases (Supplementary Figure 4). Although TIM3 and CTLA4 expression showed similar trends generally, but did not have the uniformity as PD-L1.

## Discussion

The immune microenvironment plays a pivotal role in tumor progression (Biswas, 2015; Chen and Mellman, 2017). Brooks used a 54-gene hypoxia-immune signature to identify subtype associated with prognosis and potentially responsive to targeted immunotherapies in head and neck cancer (Brooks et al., 2019). In breast cancer, Oshi et al. (2020) also generated a 4-gene score to determine the subtype which response to neoadjuvant chemotherapy and show with high expression of T cell exhaustion marker genes. The clinical significance of immune classification has been demonstrated in many diseases (Brooks et al., 2019; Li et al., 2019; Oshi et al., 2020); therefore, we attempted to explore the relationship between immunity-related genes and the clinical significance of PDAC in this study. Kandimalla et al. (2020) constructed an immune, stromal and proliferation (ISP) gene signature to predict patient outcome in PDAC. They obtained a 15-gene signature from a 170 ISP-related genes panel. However, we focused on the immune-related genes and acquired the 18 immunity-related gene signature from an 1,811 immunity-related genes panel. IL32 appeared both in ISP related signature and our immune signature, indicating that IL32 could be a pivotal molecule in PDAC progression. ISP signature and our 18 immunity-related gene signature could serve as independent predictors to predict OS of the PDAC patients, and our immune signature also showed stronger prediction capability than TNM stage. Furthermore, our results further indicated that patients in high score group PDACs could benefit from gemcitabine-based chemotherapy and patients in low score group may potentially response to PD1/PD-L1 blockade.

In this study, we firstly developed an 18 immunity-related gene classifier to predict patient outcome from TCGA data. PDAC patients with high immune score ≥20.91 were defined as high score group, and the others were defined as low score group. High score group showed shorter overall survival, which was validated in another four independent cohorts (a total of 347 patients). GO and KEGG pathway analysis revealed that the GO terms of cell cycle, cell and mitotic nuclear division were significantly enriched in high score group, such as G1/S phase transition. Besides, high score group also displayed higher levels of the proliferative indexes of Ki67 and PCNA and low expression of the gemcitabine resistance related genes. As is well known, gemcitabine mainly blocks cell cycle G1/S phase transition to exert anti-tumor activity (Fu et al., 2018). The above results indicated that high score group may be the candidate who benefited from gemcitabine-based chemotherapy. However, patients in low score group receiving chemotherapy showed no benefits and even worse prognosis.

Our signature construction was based on immunity; therefore, we explored the relationship between the subtypes and the response to immunotherapy. PD-L1 expressed on the surface of tumor cells could recognize and bind PD1 expressed on effector T cells, which transmit inhibitory immune signals to induce T cell apoptosis and inhibit T cell activation and proliferation (Gibney et al., 2016; Emens, 2018). However, the anti-PD1/PD-L1 agents in PDAC have limited efficacy (Lu et al., 2017; Mace et al., 2018). In addition to establishing effective combination therapy, it is also necessary to identify subtypes suitable for anti-PD1/PD-L1 immunotherapy (Topalian et al., 2015). In this study, patients with low PD-L1 expression suggested a favorable prognosis in low score group in two cohorts (TCGA and Ruijin). In the ICGC CA data, the result was different from other four datasets, perhaps because of microdissection which resulted in removal of immune component. In the ICGC AU and GSE 57495 cohorts, the lack of statistical differences might have been caused by the small sample size. However, in high score group, PD-L1 did not perform this function or even showed the reverse results. Furthermore, the RNA levels of CD3, CD4, and CD8 in low score group with high PD-L1 expression showed the highest level among the four subtypes in the TCGA database. GSVA analysis also indicated T cell immunoreaction activation and PD1-related immunosuppression in PD-L1(+) cases in low score group. These results were also supported by the IHC results in the Ruijin cohort, in which CD3(+), CD8(+) T cells displayed a distinct enrichment in PD-L1(+) low score group. The infiltration of CD3(+), CD8(+) T cells was evidence that could be used to predict a patient’s response to anti-PD1/PD-L1 immunotherapy (Ribas et al., 2017; Danilova et al., 2019). In this study, the proportion of patients in low score group with high PD-L1 expression was approximately 20%. This finding was consistent with previous studies that only 10–30% of patients respond to anti-PD1/PD-L1 therapy (Page et al., 2014; Ott et al., 2019). Taken together, the patients with high PD-L1 expression in low score group might be a potential subtype suitable for anti-PD1/PD-L1 immunotherapy in PDAC.

In conclusion, by analyzing genomic data from the TCGA database, we constructed an immunity-related signature to divide PDACs into two subtypes: low score and high score groups. Patients in high score group showed inferior prognosis, but could benefit from gemcitabine-based chemotherapy. Furthermore, results also indicated that PD-L1(+) tumors in low score group might respond to PD1/PD-L1 blockade therapy.

## Data Availability Statement

Raw count data and corresponding clinical characteristics of 173 patients with PDAC were downloaded from the TCGA database (https://cancergenome.nih.gov/, Project ID: TCGA-PAAD). ICGC CA (Canada) raw RNA sequencing dataset and corresponding clinical characteristics of 115 patients, and the ICGC AU (Australia) gene expression microarray dataset and corresponding clinical characteristics of 68 patients were downloaded from the ICGC database (https://icgc.org/, Code: PACA-CA and PACA-AU). The GEO dataset GSE57495 and corresponding clinical characteristics of 63 patients were downloaded from GEO database (https://www.ncbi.nlm.nih.gov/geo/).

## Ethics Statement

The studies involving human participants were reviewed and approved by the Ethical Committee of Ruijin Hospital, Shanghai Jiao Tong University School of Medicine, Shanghai, China. The patients/participants provided their written informed consent to participate in this study.

## Author Contributions

HQ, HzL, and JX developed the concept and designed the experiments. HQ wrote the manuscript. HzL, JX, FL, XT, and MS performed the experiments. WW and XL collected the patients’ information. HwL, HC, CP, ZX, XD, and BS performed the surgery. LJ, ZX, and XD helped to interpret results. BS coordinated the study and corrected the manuscript. All authors contributed to the article and approved the submitted version.

## Conflict of Interest

The authors declare that the research was conducted in the absence of any commercial or financial relationships that could be construed as a potential conflict of interest.

## Footnotes

1 https://cancer genome.nih.gov/

2 https://icgc.org/

3 https://www.ncbi.nlm.nih.gov/geo/

4 https://www.immport.org/home

5 https://www.r-project.org/

6 https://www.gsea-msigdb.org/gsea/msigdb/index.jsp

## Supplementary Material

Supplementary Figure 1 Univariate and multivariate Cox regression analysis of our immune signature and clinicopathological features in TCGA and Ruijin cohorts. Forest plots show HR and *p* value in **(A,B)** TCGA and **(C,D)** Ruijin cohorts.

Supplementary Figure 2 Pathways involved in the immunity-related 18-gene signature. The enrichment GO and KEGG pathway analyses of DEGs in **(A)** TCGA, **(B)** ICGC AU, **(C)** ICGC CA databases.

Supplementary Figure 3 Low score/PD-L1(+) PDAC showed inferior prognosis. Kaplan-Meier survival curves of PD-L1 expression in all patients, the low score group, and the high score group in **(A)** ICGC CA (*n* = 115) and **(B)** ICGC AU (*n* = 68), and **(C)** GSE57495 (*n* = 63). **(D)** PD-L1 expression between the low score and the high score group in TCGA, ICGC CA, ICGC AU, GSE57495, and Ruijin cohorts (TCGA: *p* = 0.18; ICGC CA: *p* = 0.16; ICGC AU: *p* = 0.78; GSE57495: *p* = 0.24; Ruijin: *p* = 0.16).

Supplementary Figure 4 Prognosis analysis of TIM3 and CTLA4 in ICGC CA, ICGC AU, and GSE57495 cohorts. Kaplan-Meier survival curves of TIM3 expression in all patients, or by subgroup analysis based on our immune signature in **(A)** ICGC CA (*n* = 115) and **(B)** ICGC AU (*n* = 68), and **(C)** GSE57495 (*n* = 63). Kaplan-Meier survival curves of CTLA4 expression in all patients, or by subgroup analysis based on our immune signature in **(D)** ICGC CA (*n* = 115) and **(E)** ICGC AU (*n* = 68), and **(F)** GSE57495 (*n* = 63).

Supplementary Table 1 The 1308 immunity-related genes obtained by Cox univariate analysis.

Supplementary Table 2 The risk score formula of immune-related 18-gene signature.

Supplementary Table 3 Sequences of the qRT-PCR primers used in this study.

Supplementary Table 4 The DEGs between the gemcitabine resistance population and main tumor cell population in GSE36563 dataset.

Supplementary Table 5 The gene name of each row from the heatmap in Figure 7B.
