# Supplementary figures and images for: Immunity-Related Gene Signature Identifies Subtypes Benefitting From Adjuvant Chemotherapy or Potentially Responding to PD1/PD-L1 Blockage in Pancreatic Cancer
Source: Front Cell Dev Biol. 2021 Jun 23;9:682261. doi: 10.3389/fcell.2021.682261 (PMC8264789; doi:10.3389/fcell.2021.682261)

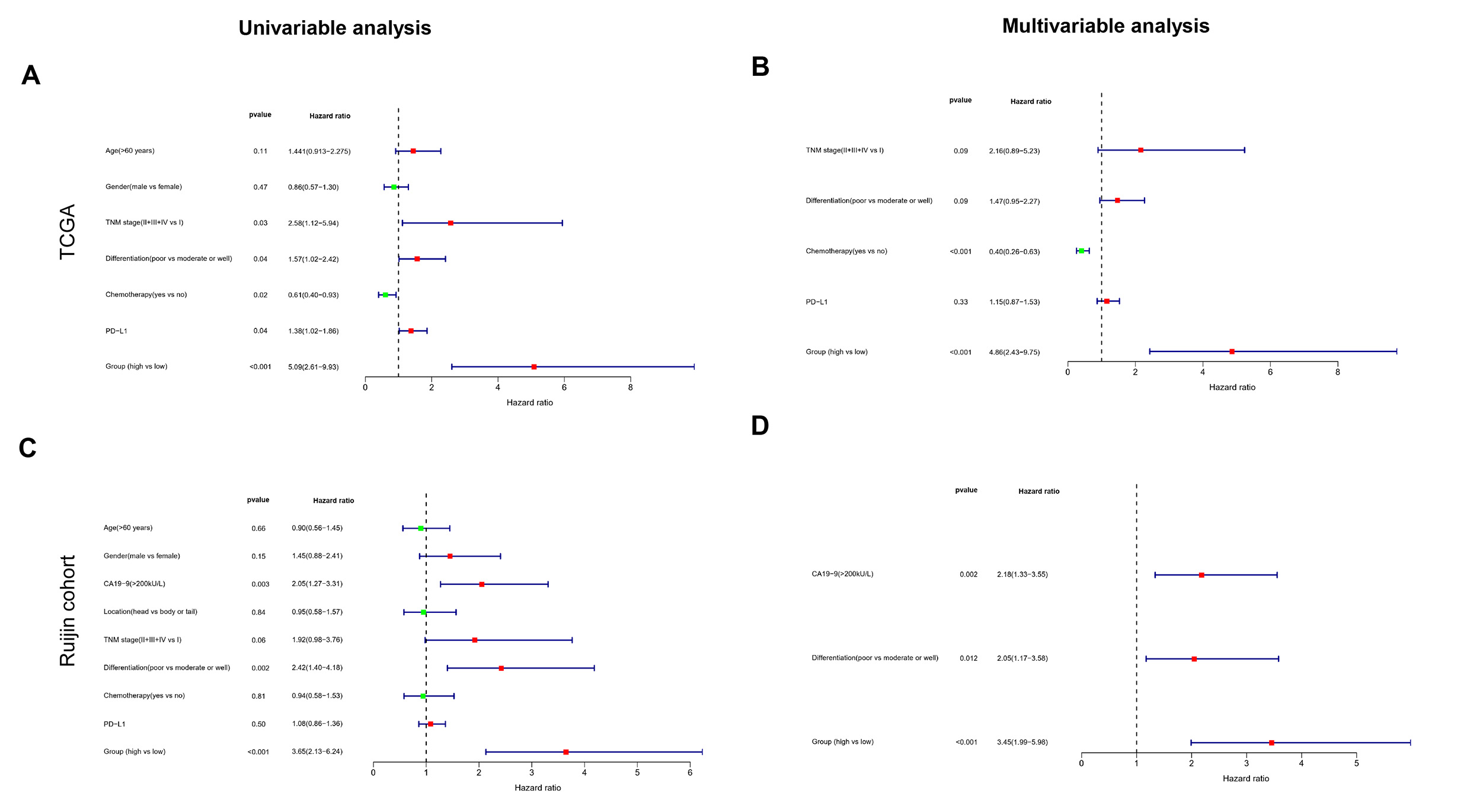

Supplement: Supplementary Figure 1 — Univariate and multivariate Cox regression analysis of our immune signature and clinicopathological features in TCGA and Ruijin cohorts. Forest plots show HR and p value in (A,B) TCGA and (C,D) Ruijin cohorts. [file Image_1.JPEG]

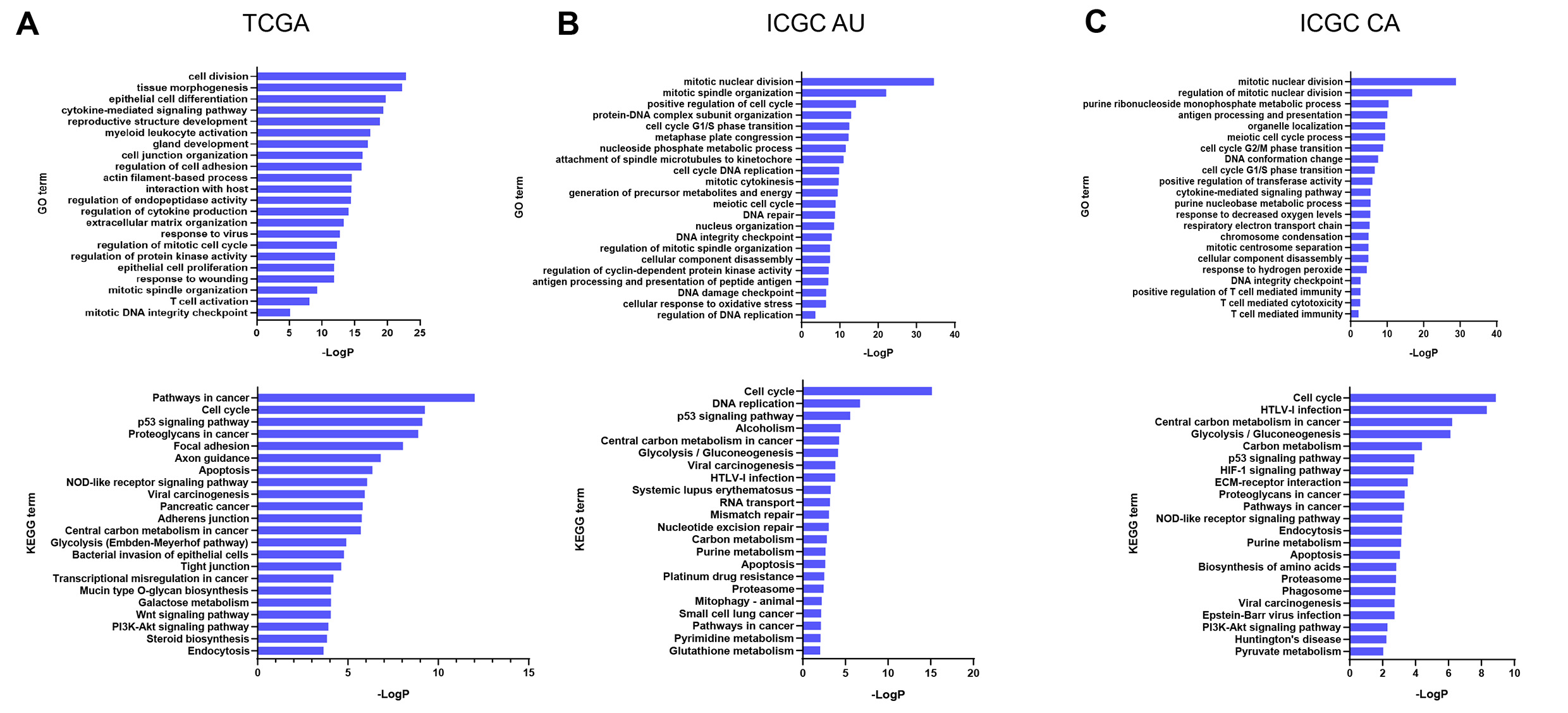

Supplement: Supplementary Figure 2 — Pathways involved in the immunity-related 18-gene signature. The enrichment GO and KEGG pathway analyses of DEGs in (A) TCGA, (B) ICGC AU, (C) ICGC CA databases. [file Image_2.JPEG]

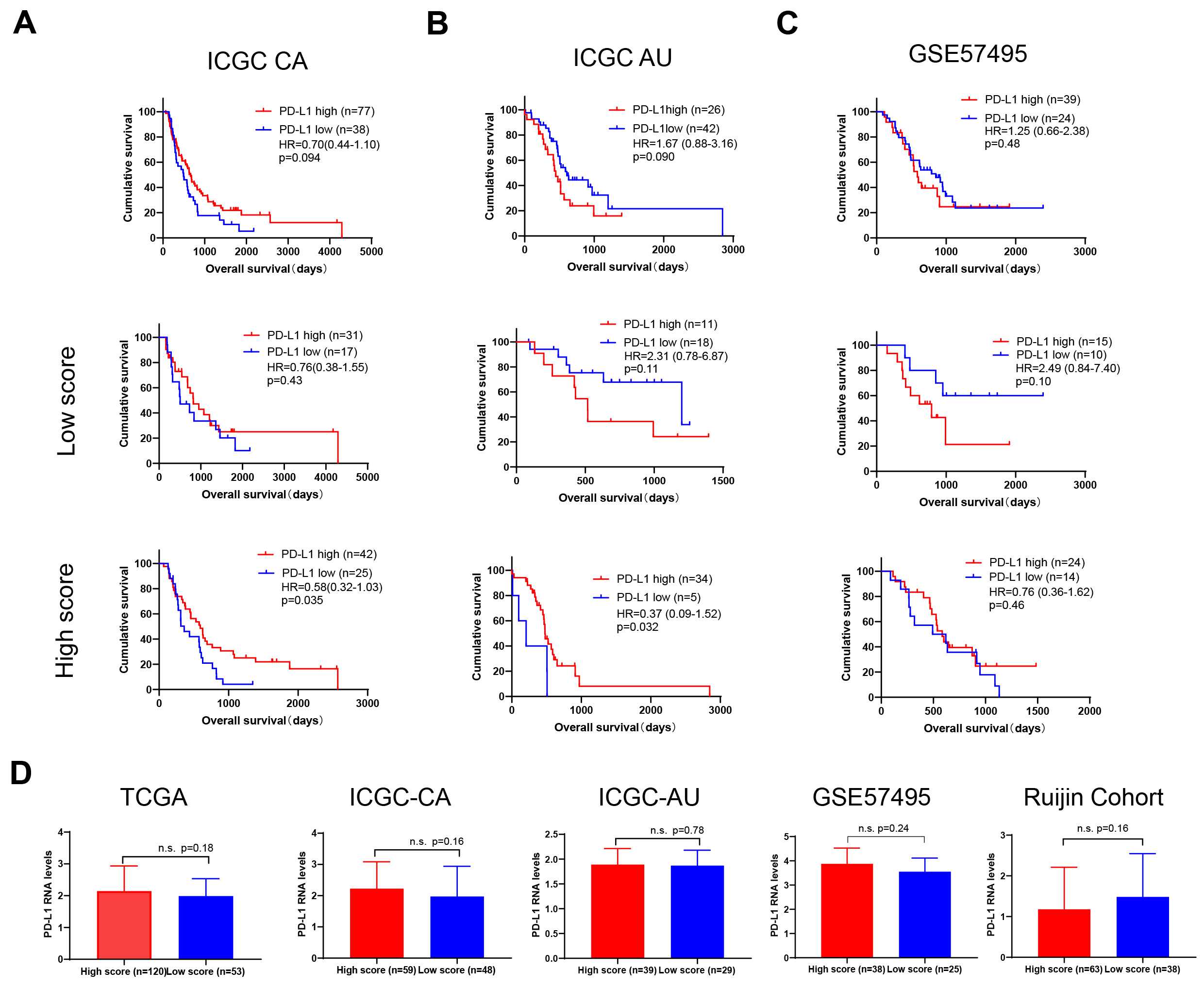

Supplement: Supplementary Figure 3 — Low score/PD-L1(+) PDAC showed inferior prognosis. Kaplan-Meier survival curves of PD-L1 expression in all patients, the low score group, and the high score group in (A) ICGC CA (n = 115) and (B) ICGC AU (n = 68), and (C) GSE57495 (n = 63). (D) PD-L1 expression between the low score and the high score group in TCGA, ICGC CA, ICGC AU, GSE57495, and Ruijin cohorts (TCGA: p = 0.18; ICGC CA: p = 0.16; ICGC AU: p = 0.78; GSE57495: p = 0.24; Ruijin: p = 0.16). [file Image_3.JPEG]

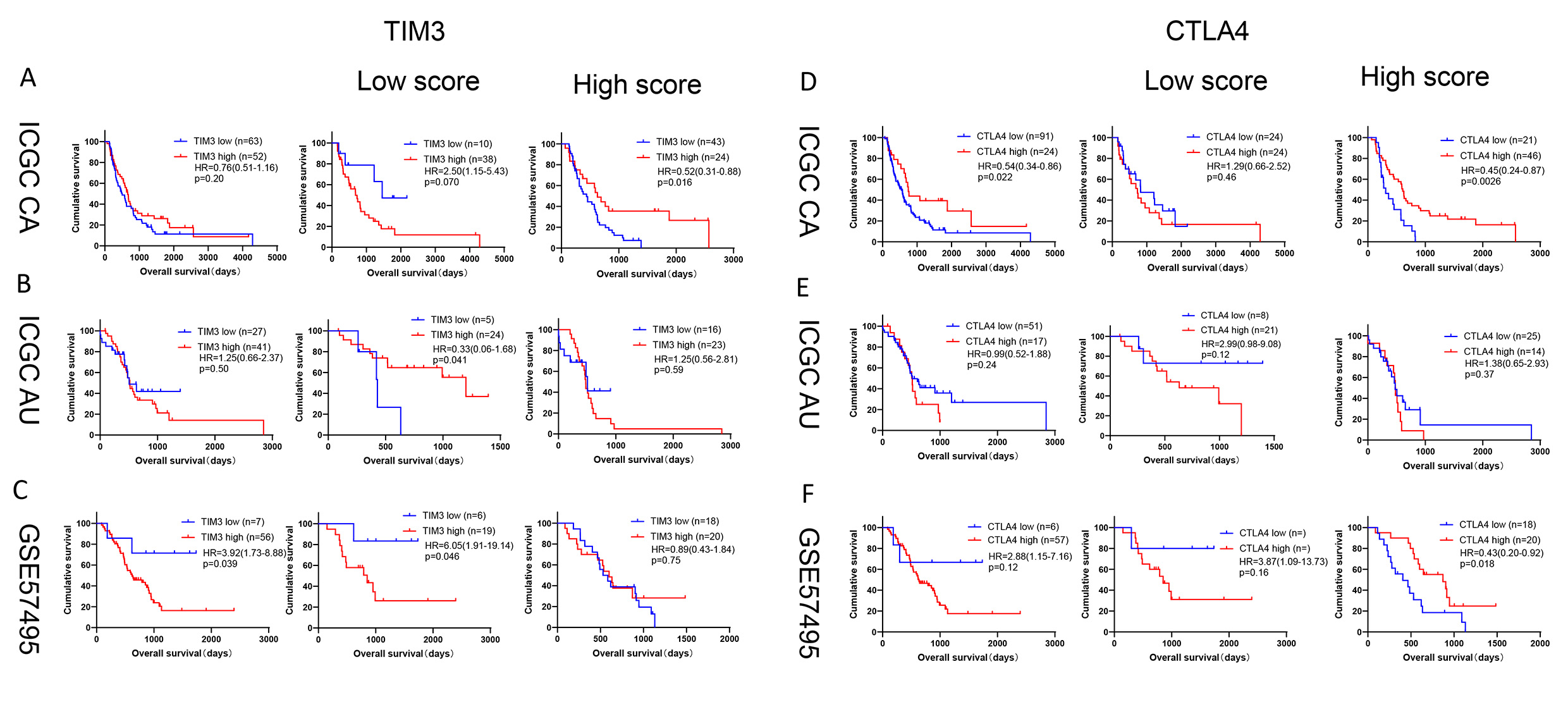

Supplement: Supplementary Figure 4 — Prognosis analysis of TIM3 and CTLA4 in ICGC CA, ICGC AU, and GSE57495 cohorts. Kaplan-Meier survival curves of TIM3 expression in all patients, or by subgroup analysis based on our immune signature in (A) ICGC CA (n = 115) and (B) ICGC AU (n = 68), and (C) GSE57495 (n = 63). Kaplan-Meier survival curves of CTLA4 expression in all patients, or by subgroup analysis based on our immune signature in (D) ICGC CA (n = 115) and (E) ICGC AU (n = 68), and (F) GSE57495 (n = 63). [file Image_4.JPEG]
