# Supplementary material for: Immunity-Related Gene Signature Identifies Subtypes Benefitting From Adjuvant Chemotherapy or Potentially Responding to PD1/PD-L1 Blockage in Pancreatic Cancer
Source: Front Cell Dev Biol. 2021 Jun 23;9:682261. doi: 10.3389/fcell.2021.682261 (PMC8264789; doi:10.3389/fcell.2021.682261)
Supplement: Supplementary Table 1 — The 1308 immunity-related genes obtained by Cox univariate analysis. [file Table_1.DOCX]

Supplemental Table 1. The 1308 immunity-related genes obtained by Cox univariate analysis

| Gene name | pvalue | HR |
| --- | --- | --- |
| S100A2 | 0.000102 | 1.476843 |
| PSPN | 0.000102 | 0.634199 |
| OAS1 | 0.000102 | 1.536421 |
| PLAU | 0.000105 | 1.625449 |
| DKK1 | 0.000109 | 1.520346 |
| SLURP1 | 0.000114 | 1.415876 |
| GNRH2 | 0.000118 | 0.606808 |
| EREG | 0.000123 | 1.468587 |
| S100P | 0.000146 | 1.70156 |
| GBP2 | 0.000159 | 1.735892 |
| IL1RN | 0.000161 | 1.586922 |
| KCNH2 | 0.00017 | 0.648513 |
| SLC22A17 | 0.000177 | 0.632469 |
| IL15RA | 0.000194 | 1.607299 |
| S100A14 | 0.000205 | 1.603276 |
| SDC4 | 0.00023 | 1.586813 |
| FGF17 | 0.000233 | 0.572778 |
| CKLF | 0.000242 | 1.573074 |
| S100A11 | 0.000254 | 1.7027 |
| S100A10 | 0.000267 | 1.553854 |
| PRLR | 0.000268 | 0.593293 |
| BIRC5 | 0.000305 | 1.460327 |
| DEFB116 | 0.000305 | 1.496586 |
| SEMA3C | 0.000308 | 1.509607 |
| CXCL11 | 0.000342 | 1.414735 |
| AREG | 0.000362 | 1.468501 |
| PSMB8 | 0.000366 | 1.605633 |
| JAG1 | 0.000386 | 1.522352 |
| IL1RAP | 0.000392 | 1.500493 |
| TAP2 | 0.000407 | 1.484928 |
| FAM19A2 | 0.000422 | 0.504518 |
| TNFRSF10A | 0.000482 | 1.478916 |
| IL18 | 0.000487 | 1.608842 |
| TNFRSF12A | 0.000499 | 1.518296 |
| DEFB114 | 0.00051 | 1.3521 |
| DEFB113 | 0.00051 | 1.5047 |
| IL1R2 | 0.000511 | 1.450172 |
| FGFRL1 | 0.000533 | 1.439538 |
| NCK1 | 0.000559 | 1.581787 |
| TAP1 | 0.000565 | 1.513891 |
| FIGNL2 | 0.000601 | 0.489168 |
| CRABP2 | 0.000608 | 1.47169 |
| IL32 | 0.000626 | 1.543479 |
| PIK3CB | 0.000654 | 1.491696 |
| WFIKKN1 | 0.000694 | 0.662819 |
| FAM3C | 0.000755 | 1.482489 |
| CMTM1 | 0.000792 | 1.433248 |
| RFXAP | 0.000844 | 0.702286 |
| NRAS | 0.000859 | 1.510992 |
| GDF9 | 0.000869 | 0.625559 |
| RLN2 | 0.000887 | 0.678231 |
| RLN1 | 0.000905 | 0.656043 |
| TRIM5 | 0.000909 | 1.517946 |
| AHNAK | 0.000915 | 1.510548 |
| TMSB10 | 0.00092 | 1.483876 |
| CHGA | 0.000927 | 0.710503 |
| SP1 | 0.001011 | 1.498932 |
| STAT1 | 0.001125 | 1.432818 |
| PAK2 | 0.001149 | 1.504021 |
| BST2 | 0.00116 | 1.428882 |
| PPARG | 0.00117 | 1.393502 |
| PLAUR | 0.001172 | 1.501381 |
| IL22RA1 | 0.001262 | 1.491102 |
| TNFSF9 | 0.001282 | 1.371876 |
| NPPA | 0.001288 | 0.678982 |
| DEFB1 | 0.00133 | 1.382468 |
| PDGFC | 0.001332 | 1.492738 |
| VTN | 0.001459 | 0.692584 |
| CXCL10 | 0.00156 | 1.380703 |
| SEMA6C | 0.001658 | 0.744689 |
| HDGF | 0.001697 | 1.455277 |
| SCG2 | 0.001805 | 0.711838 |
| CXCL17 | 0.00182 | 1.392649 |
| PML | 0.001821 | 1.499071 |
| OSMR | 0.00191 | 1.450456 |
| TNFRSF21 | 0.001957 | 1.516417 |
| KLKB1 | 0.001966 | 0.692116 |
| CCRL1 | 0.001985 | 1.386641 |
| VDR | 0.002009 | 1.46065 |
| GDF11 | 0.002011 | 0.733518 |
| TSHB | 0.002017 | 0.63338 |
| S100A6 | 0.00205 | 1.466841 |
| PLSCR1 | 0.002057 | 1.458641 |
| PSME1 | 0.002063 | 1.462852 |
| NRG2 | 0.002099 | 0.682001 |
| SDC1 | 0.002107 | 1.415737 |
| IFITM1 | 0.002189 | 1.385738 |
| TNFRSF10B | 0.002211 | 1.36832 |
| INSL5 | 0.002236 | 0.494379 |
| PI3 | 0.002331 | 1.384266 |
| TMSB4X | 0.002352 | 1.507721 |
| EGFR | 0.002476 | 1.356439 |
| CLDN4 | 0.00248 | 1.456121 |
| CTSE | 0.002551 | 1.44434 |
| LRSAM1 | 0.002592 | 0.760424 |
| CGB7 | 0.002595 | 1.335478 |
| PPIA | 0.002723 | 1.456393 |
| ANGPTL5 | 0.002807 | 0.654874 |
| S100A13 | 0.002819 | 1.434771 |
| TYK2 | 0.002824 | 0.765445 |
| PPP3CA | 0.002914 | 1.421624 |
| TLR3 | 0.002938 | 1.381746 |
| TCF7L2 | 0.003026 | 1.38386 |
| KL | 0.00309 | 0.665588 |
| SSTR2 | 0.003095 | 0.659514 |
| SHC2 | 0.003102 | 0.751814 |
| RXRA | 0.003104 | 0.750343 |
| CLCF1 | 0.003139 | 1.368809 |
| PCSK2 | 0.003197 | 0.729234 |
| GIPR | 0.003238 | 0.741472 |
| AGT | 0.003281 | 0.703962 |
| CCR10 | 0.003356 | 0.677965 |
| CHGB | 0.003371 | 0.744511 |
| ERAP2 | 0.003424 | 1.398232 |
| PTGS2 | 0.003436 | 1.348968 |
| TFRC | 0.003456 | 1.405078 |
| F2RL1 | 0.003504 | 1.372057 |
| TINAGL1 | 0.003506 | 1.379517 |
| CHP | 0.003589 | 1.366984 |
| GDF1 | 0.003603 | 0.657531 |
| ITGAV | 0.0037 | 1.396117 |
| MAPT | 0.003763 | 0.65946 |
| UCN3 | 0.003777 | 0.730063 |
| HBEGF | 0.003931 | 1.351368 |
| PRDX1 | 0.003933 | 1.432029 |
| PAK3 | 0.003993 | 0.723212 |
| GPR77 | 0.004033 | 1.350533 |
| POMC | 0.004065 | 0.677446 |
| IFIH1 | 0.004109 | 1.35901 |
| MSTN | 0.004119 | 0.683829 |
| VGF | 0.004283 | 0.73379 |
| NENF | 0.004324 | 0.721426 |
| NTF4 | 0.004388 | 1.303184 |
| SEMA4B | 0.004389 | 1.365085 |
| SEMA3G | 0.004392 | 0.744061 |
| WNT5A | 0.004456 | 1.360586 |
| LTB4R | 0.004665 | 0.742878 |
| AGER | 0.004686 | 0.743948 |
| IL11RA | 0.004714 | 0.738618 |
| RAC1 | 0.00472 | 1.386672 |
| MC1R | 0.00474 | 0.753265 |
| LEAP2 | 0.004867 | 0.73216 |
| CXCL9 | 0.004954 | 1.303594 |
| INHBA | 0.00498 | 1.386258 |
| SPP1 | 0.00505 | 1.359576 |
| HFE | 0.005056 | 1.397658 |
| IL17RE | 0.005104 | 1.351643 |
| NR0B1 | 0.00512 | 0.712447 |
| NAMPT | 0.00517 | 1.381076 |
| CST4 | 0.005283 | 1.307477 |
| SEMA7A | 0.005285 | 1.356973 |
| ISG15 | 0.005324 | 1.332955 |
| FGF22 | 0.005374 | 0.708841 |
| FIGF | 0.005405 | 0.653759 |
| CXCL5 | 0.005514 | 1.317745 |
| BCL10 | 0.005529 | 1.346804 |
| LCN2 | 0.005607 | 1.375945 |
| TMPRSS6 | 0.005657 | 0.71547 |
| PRKCG | 0.005788 | 0.736074 |
| FGF23 | 0.005877 | 1.242909 |
| GKN1 | 0.005931 | 1.288542 |
| LMBR1L | 0.005986 | 0.768638 |
| FGF2 | 0.006019 | 1.337625 |
| UNC93B1 | 0.006108 | 1.316151 |
| RSAD2 | 0.006119 | 1.364061 |
| KRAS | 0.006345 | 1.382342 |
| FAM19A4 | 0.006361 | 0.480523 |
| S100A3 | 0.006392 | 1.326133 |
| GH1 | 0.006414 | 0.632016 |
| C20orf186 | 0.006585 | 0.214077 |
| IL11 | 0.00665 | 1.294866 |
| PSMD2 | 0.007134 | 1.388971 |
| PSME2 | 0.007272 | 1.362124 |
| APOBEC3C | 0.007299 | 1.370606 |
| CXCR7 | 0.00738 | 1.347728 |
| PCSK1 | 0.007502 | 0.736936 |
| PAK7 | 0.007579 | 0.718776 |
| CD320 | 0.007736 | 0.749997 |
| UCN2 | 0.00777 | 1.304557 |
| SSTR1 | 0.007817 | 0.752971 |
| FGF14 | 0.00792 | 0.733578 |
| RARG | 0.007945 | 1.346946 |
| TACR1 | 0.008153 | 0.725223 |
| TOR2A | 0.008535 | 0.756171 |
| NPR1 | 0.008739 | 0.74451 |
| SFTPA2 | 0.008739 | 1.26926 |
| LCN12 | 0.00875 | 0.763332 |
| SLPI | 0.008824 | 1.365213 |
| AMH | 0.008828 | 0.757681 |
| IL6R | 0.009158 | 0.771288 |
| IL13RA2 | 0.00916 | 0.693017 |
| PSMD14 | 0.009363 | 1.340356 |
| PAK6 | 0.009372 | 0.68995 |
| APOH | 0.009383 | 0.76593 |
| ARG2 | 0.009474 | 0.726832 |
| EPOR | 0.009481 | 0.776894 |
| KITLG | 0.009686 | 1.32379 |
| ADM2 | 0.009745 | 1.304454 |
| FAM19A1 | 0.009781 | 0.701087 |
| CTSB | 0.010041 | 1.329616 |
| DEFB123 | 0.010266 | 1.274916 |
| EDNRA | 0.01028 | 1.344454 |
| CGB8 | 0.010308 | 1.28069 |
| DEFB115 | 0.010317 | 1.223086 |
| FGF9 | 0.010421 | 0.754301 |
| GPR44 | 0.010477 | 0.743516 |
| LIMS1 | 0.010604 | 1.348582 |
| MPL | 0.010829 | 0.706425 |
| CBLC | 0.011155 | 1.348328 |
| B2M | 0.011176 | 1.374168 |
| S100Z | 0.011511 | 0.724354 |
| SEMA3B | 0.011726 | 1.290454 |
| NRP2 | 0.011742 | 1.336786 |
| MUC4 | 0.011869 | 1.280614 |
| IL31RA | 0.012099 | 1.210259 |
| FGF12 | 0.012334 | 0.753788 |
| IL4 | 0.012591 | 0.65246 |
| PIK3CA | 0.012849 | 1.327051 |
| PRDX2 | 0.012865 | 0.770292 |
| ADRB1 | 0.012964 | 0.747959 |
| GPER | 0.013223 | 0.758572 |
| AVPR1B | 0.013415 | 0.688836 |
| MUC5AC | 0.013504 | 1.277967 |
| LTBP1 | 0.01353 | 1.334152 |
| ANGPTL3 | 0.013847 | 0.714765 |
| BMP4 | 0.014035 | 1.280144 |
| TRAF3 | 0.014107 | 0.775453 |
| RAC2 | 0.014241 | 1.303522 |
| KLRC2 | 0.014477 | 0.669279 |
| MAP3K14 | 0.014651 | 0.766756 |
| SERPIND1 | 0.014673 | 0.744628 |
| CMTM6 | 0.014698 | 1.340508 |
| GREM1 | 0.014902 | 1.301286 |
| RELA | 0.014908 | 1.31981 |
| SLIT1 | 0.014947 | 0.72951 |
| LHB | 0.015109 | 1.263607 |
| PGLYRP3 | 0.015186 | 1.258919 |
| FGF8 | 0.015264 | 1.280653 |
| FABP12 | 0.01534 | 0.697901 |
| GRAP2 | 0.015767 | 0.749288 |
| CDH1 | 0.016463 | 1.361119 |
| IL3RA | 0.01654 | 0.773753 |
| CGB5 | 0.016656 | 1.255907 |
| RBP5 | 0.016947 | 0.761051 |
| FAS | 0.016957 | 1.287214 |
| TEC | 0.017181 | 1.28453 |
| MASP2 | 0.017485 | 0.753717 |
| TNFRSF10D | 0.017632 | 1.24571 |
| GHR | 0.017714 | 0.758962 |
| FYN | 0.018806 | 0.773818 |
| NOX4 | 0.018988 | 1.292738 |
| IL17D | 0.019006 | 0.766835 |
| GSK3B | 0.019387 | 1.34382 |
| CCL28 | 0.019552 | 1.276184 |
| DDX58 | 0.020329 | 1.274008 |
| IL20RA | 0.020469 | 1.304021 |
| MICB | 0.02048 | 1.269608 |
| PSMD1 | 0.020695 | 1.323841 |
| SRC | 0.020985 | 1.311579 |
| PTHLH | 0.02128 | 1.255172 |
| VCAM1 | 0.021574 | 1.295505 |
| CARD11 | 0.021968 | 1.271607 |
| TYMP | 0.022134 | 1.281697 |
| IL8 | 0.022175 | 1.261037 |
| CD40 | 0.022199 | 1.306064 |
| S100A1 | 0.023197 | 0.772682 |
| PAK1 | 0.023642 | 1.287334 |
| LTB4R2 | 0.023968 | 0.778389 |
| MARCO | 0.02399 | 1.260278 |
| SEMA6D | 0.024202 | 0.78646 |
| EIF2AK2 | 0.024433 | 1.307324 |
| S100B | 0.024624 | 0.805274 |
| CRHR2 | 0.024827 | 0.72437 |
| LGR4 | 0.024858 | 1.275921 |
| IL29 | 0.025033 | 1.244078 |
| RABEP1 | 0.025061 | 0.801508 |
| ADM | 0.025303 | 1.251053 |
| BID | 0.025339 | 1.272945 |
| VIPR1 | 0.025415 | 0.789626 |
| LIFR | 0.025461 | 0.792242 |
| RABEP2 | 0.025826 | 0.802002 |
| TNFRSF4 | 0.025963 | 0.790866 |
| MAVS | 0.026457 | 0.795925 |
| MTNR1B | 0.026534 | 0.738844 |
| DEFB127 | 0.026542 | 1.176267 |
| IL28A | 0.027835 | 1.234471 |
| LAT | 0.028032 | 0.760547 |
| EDN2 | 0.028442 | 1.232477 |
| LYN | 0.028651 | 1.268052 |
| NMBR | 0.028969 | 1.282773 |
| PPARD | 0.029102 | 1.269443 |
| GPR17 | 0.029198 | 0.769457 |
| TGFB2 | 0.029212 | 1.251919 |
| TNFSF11 | 0.029322 | 1.262888 |
| PPP3R1 | 0.029451 | 1.330638 |
| IL28B | 0.029705 | 1.233808 |
| DEFB134 | 0.029741 | 0.770008 |
| BMP2 | 0.029882 | 1.275204 |
| MR1 | 0.030474 | 1.266198 |
| CGA | 0.030653 | 0.587549 |
| CETP | 0.031031 | 0.75449 |
| SHC1 | 0.031438 | 1.260164 |
| LTBP2 | 0.031464 | 1.278593 |
| BRD8 | 0.031906 | 0.819923 |
| NRTN | 0.033434 | 0.796347 |
| FGF13 | 0.033451 | 0.773602 |
| IRF1 | 0.033512 | 1.305468 |
| IFNW1 | 0.033804 | 0.743541 |
| RXRG | 0.034235 | 0.785071 |
| PSMD13 | 0.034374 | 1.264074 |
| IL27 | 0.034571 | 1.256498 |
| GHRHR | 0.035116 | 0.740983 |
| SEMA4D | 0.035228 | 0.79893 |
| RHOA | 0.03586 | 1.294095 |
| IL4R | 0.03605 | 1.256598 |
| C3 | 0.036569 | 1.238824 |
| C5 | 0.036731 | 0.784631 |
| CTSS | 0.036771 | 1.235518 |
| NFYA | 0.03715 | 1.262469 |
| ANXA6 | 0.037475 | 0.814772 |
| CXCL6 | 0.037598 | 1.256693 |
| RLN3 | 0.03849 | 0.759081 |
| S100A9 | 0.038608 | 1.233853 |
| CSPG5 | 0.038664 | 0.794414 |
| ANGPTL4 | 0.038775 | 1.225937 |
| PLXNA1 | 0.039827 | 1.24661 |
| EGF | 0.039951 | 1.233523 |
| ADIPOR1 | 0.041701 | 1.256307 |
| HLA-B | 0.041941 | 1.245141 |
| PRLHR | 0.042313 | 0.700159 |
| DLL4 | 0.042328 | 0.816029 |
| STAB2 | 0.042887 | 0.775056 |
| CMTM3 | 0.04328 | 1.271637 |
| CCRL2 | 0.043458 | 1.236891 |
| NOD2 | 0.043533 | 1.218409 |
| LGR6 | 0.043752 | 1.225227 |
| BMP1 | 0.04382 | 1.245449 |
| PSMD7 | 0.044261 | 1.277358 |
| OPRD1 | 0.045144 | 0.73455 |
| NR4A2 | 0.045735 | 1.244759 |
| GDF7 | 0.045791 | 0.790755 |
| FLT4 | 0.046107 | 0.814197 |
| AGTR1 | 0.046141 | 0.798851 |
| CDC42 | 0.046144 | 1.271581 |
| C8G | 0.047836 | 0.785118 |
| KAL1 | 0.048169 | 1.219717 |
| NGFR | 0.048397 | 0.811949 |
| RBP7 | 0.048849 | 0.80263 |
| NFAT5 | 0.049256 | 1.261543 |
| PLCG1 | 0.049416 | 0.830049 |
| LCN10 | 0.050424 | 0.765648 |
| ESRRB | 0.050499 | 0.798426 |
| LIF | 0.050762 | 1.217062 |
| HSPA1L | 0.051617 | 0.810859 |
| EDN1 | 0.053579 | 1.217314 |
| CSF2 | 0.053992 | 1.199529 |
| GHRL | 0.054542 | 0.798664 |
| IL17B | 0.054891 | 0.766906 |
| TNFSF4 | 0.054991 | 1.243938 |
| PPP3CB | 0.055212 | 0.826274 |
| GZMB | 0.055226 | 1.221859 |
| PRTN3 | 0.055233 | 0.733447 |
| NR1H3 | 0.056554 | 1.224229 |
| ZYX | 0.057698 | 1.23764 |
| SLC11A1 | 0.057813 | 1.219052 |
| OLR1 | 0.057835 | 1.205485 |
| CRHR1 | 0.058126 | 0.789241 |
| SYK | 0.058292 | 1.219677 |
| FCGR2B | 0.059534 | 1.219256 |
| IL23A | 0.059663 | 1.211095 |
| TLR1 | 0.059674 | 1.20551 |
| ARMETL1 | 0.06011 | 0.820213 |
| PPP4C | 0.061172 | 1.2439 |
| EDN3 | 0.061301 | 0.824001 |
| PDK1 | 0.061508 | 1.205148 |
| CAT | 0.061591 | 1.214756 |
| LTBR | 0.062431 | 1.19297 |
| IL13 | 0.062468 | 0.750012 |
| ELANE | 0.062589 | 0.727629 |
| SSTR5 | 0.062737 | 0.817131 |
| NRG1 | 0.062785 | 0.770515 |
| ISG20 | 0.062911 | 1.216746 |
| IFNA21 | 0.06322 | 0.749661 |
| IFNGR1 | 0.063387 | 1.242502 |
| HDGFRP3 | 0.063822 | 0.832785 |
| PI15 | 0.064645 | 1.222293 |
| DARC | 0.065324 | 0.816729 |
| DUOX2 | 0.065334 | 1.212838 |
| CNTF | 0.065432 | 1.205866 |
| HNF4G | 0.065492 | 1.208979 |
| TXLNA | 0.065543 | 0.83289 |
| IL10RB | 0.066446 | 1.207644 |
| CER1 | 0.066828 | 0.640685 |
| SECTM1 | 0.066913 | 1.207736 |
| CECR1 | 0.067597 | 0.837408 |
| LPA | 0.069612 | 0.804094 |
| ICAM1 | 0.069625 | 1.211746 |
| IFNAR1 | 0.069836 | 1.229925 |
| FCGR3A | 0.069953 | 1.207987 |
| NMB | 0.070224 | 1.203025 |
| THBS1 | 0.070257 | 1.23032 |
| NFKBIE | 0.070832 | 1.199341 |
| CCL20 | 0.070959 | 1.175125 |
| THRA | 0.070961 | 0.828904 |
| HLA-F | 0.072241 | 1.213388 |
| TMEM173 | 0.072458 | 1.222545 |
| CNTFR | 0.072756 | 0.808737 |
| PROCR | 0.073569 | 1.221707 |
| BMPR2 | 0.073598 | 1.229028 |
| NRG4 | 0.073737 | 1.198142 |
| FCER1G | 0.074126 | 1.208353 |
| IL1F6 | 0.07416 | 1.207368 |
| PF4 | 0.074285 | 1.211316 |
| PSMC4 | 0.07429 | 1.234317 |
| RBP1 | 0.075868 | 1.217747 |
| ECD | 0.075882 | 1.221774 |
| MAP2K2 | 0.075935 | 0.833241 |
| MMP9 | 0.07649 | 1.203986 |
| KLRC3 | 0.077812 | 0.784459 |
| TRIM22 | 0.078713 | 1.211781 |
| MAPK1 | 0.07884 | 1.236941 |
| PLXNB1 | 0.079949 | 0.840816 |
| BDNF | 0.080237 | 1.176702 |
| IL1F7 | 0.080364 | 1.192418 |
| TNFSF13 | 0.080788 | 1.201965 |
| RNASE2 | 0.081275 | 1.18914 |
| PSME3 | 0.081311 | 1.245776 |
| CCR7 | 0.081728 | 0.818973 |
| SEMA4G | 0.081802 | 1.201633 |
| LCN15 | 0.081892 | 0.647697 |
| ACTG1 | 0.082577 | 1.239417 |
| TNFRSF11B | 0.082709 | 1.191701 |
| NR3C2 | 0.08335 | 0.829198 |
| HLA-DMA | 0.084319 | 1.196376 |
| SEMA6A | 0.084571 | 0.827126 |
| PROK1 | 0.085253 | 0.778357 |
| HLA-C | 0.085512 | 1.203385 |
| JUND | 0.085551 | 0.841593 |
| RAET1E | 0.08637 | 1.184297 |
| TNFRSF1A | 0.086591 | 1.205959 |
| S100A8 | 0.08694 | 1.19882 |
| IL1R1 | 0.087457 | 1.200641 |
| FSHB | 0.087475 | 0.691436 |
| HLA-DRB5 | 0.089893 | 1.189379 |
| GAST | 0.090486 | 0.819433 |
| CACYBP | 0.092015 | 1.198362 |
| CXCL14 | 0.092762 | 1.197929 |
| ANGPT4 | 0.092871 | 1.184656 |
| HLA-E | 0.093139 | 1.206662 |
| IFNG | 0.093524 | 1.164405 |
| TGFBR1 | 0.093626 | 1.222291 |
| PDGFRB | 0.09385 | 1.214585 |
| LOC100129216 | 0.093981 | 1.194075 |
| MSR1 | 0.094425 | 1.184355 |
| BMP6 | 0.09487 | 0.83399 |
| TGFBR2 | 0.095406 | 1.194457 |
| S100A5 | 0.095848 | 1.164743 |
| BMP15 | 0.096477 | 1.144632 |
| IL7 | 0.096862 | 1.189728 |
| APOBEC3G | 0.097623 | 1.192412 |
| HLA-DRB1 | 0.097814 | 1.190025 |
| IL2RG | 0.098005 | 1.182248 |
| BMPR1B | 0.098101 | 1.202255 |
| BPIL1 | 0.098231 | 0.733216 |
| OSGIN1 | 0.098631 | 1.166721 |
| PSMD6 | 0.098911 | 1.193293 |
| PIK3R3 | 0.099468 | 0.842477 |
| PLTP | 0.099828 | 1.180639 |
| PSMD11 | 0.099866 | 1.202435 |
| PGC | 0.101678 | 1.182596 |
| CRLF2 | 0.102122 | 0.811798 |
| OSM | 0.102243 | 1.170006 |
| TNFSF13B | 0.102644 | 1.1756 |
| HSPA1A | 0.102779 | 1.168513 |
| KLRK1 | 0.102993 | 0.802463 |
| SEMA3F | 0.10306 | 1.193894 |
| PTAFR | 0.103089 | 1.183086 |
| PSMC3 | 0.103137 | 1.19197 |
| PSMD5 | 0.103506 | 1.214401 |
| NFATC1 | 0.104304 | 0.832988 |
| PGLYRP4 | 0.105655 | 1.171219 |
| FAM19A3 | 0.1069 | 0.838853 |
| REG1A | 0.107396 | 1.190527 |
| MC3R | 0.107718 | 0.807784 |
| CX3CL1 | 0.10873 | 1.180936 |
| OGFR | 0.109502 | 0.848678 |
| NEDD4 | 0.109632 | 1.196147 |
| PROC | 0.109807 | 0.846888 |
| ADIPOQ | 0.109832 | 0.813426 |
| CCL13 | 0.110068 | 1.187318 |
| ZAP70 | 0.110073 | 0.827698 |
| IDO1 | 0.110878 | 1.188306 |
| NTS | 0.111708 | 0.817767 |
| ANGPTL2 | 0.11231 | 1.186445 |
| SH3BP2 | 0.112752 | 1.178697 |
| TNFRSF11A | 0.116169 | 1.161341 |
| JAK1 | 0.116887 | 1.20129 |
| KDR | 0.117167 | 0.855058 |
| CMTM8 | 0.117217 | 0.854025 |
| XCR1 | 0.117831 | 0.840819 |
| DHX58 | 0.118194 | 1.188345 |
| TK2 | 0.118757 | 1.18878 |
| NR1I2 | 0.11967 | 1.181137 |
| NR2E1 | 0.120278 | 1.152731 |
| MDK | 0.120425 | 1.191119 |
| ADRB2 | 0.120447 | 0.847919 |
| CMTM5 | 0.121799 | 0.811425 |
| ULBP3 | 0.122478 | 1.145905 |
| CRIM1 | 0.122857 | 1.191382 |
| HMGB1 | 0.12309 | 1.207425 |
| PDIA2 | 0.124009 | 1.17565 |
| RAC3 | 0.124182 | 0.853763 |
| AQP9 | 0.126143 | 1.168006 |
| RELB | 0.129152 | 1.164016 |
| PDIA3 | 0.129911 | 1.19025 |
| SEMA4F | 0.130772 | 0.857399 |
| PDGFRA | 0.130999 | 1.170283 |
| CXCL3 | 0.131858 | 1.158947 |
| PROK2 | 0.131924 | 1.164792 |
| DEFB118 | 0.132235 | 1.152811 |
| ESR2 | 0.132743 | 0.846091 |
| HLA-DRA | 0.134 | 1.167159 |
| FLT3LG | 0.134294 | 0.855083 |
| CD74 | 0.134322 | 1.17106 |
| MAPK14 | 0.135533 | 1.1756 |
| PTPN6 | 0.135704 | 0.857225 |
| NPR3 | 0.135766 | 1.181001 |
| TANK | 0.135859 | 1.195378 |
| ITGB2 | 0.136111 | 1.167416 |
| RAF1 | 0.13642 | 1.187635 |
| SEMA5A | 0.136918 | 0.857991 |
| SEMA3A | 0.13746 | 1.178849 |
| IFNAR2 | 0.137582 | 1.176893 |
| FGFR1 | 0.138213 | 0.859225 |
| INHBB | 0.13843 | 1.164034 |
| FGFR2 | 0.139565 | 1.15648 |
| OSTN | 0.139946 | 0.81959 |
| OPRL1 | 0.1401 | 0.866599 |
| CCL7 | 0.144051 | 1.167692 |
| LRP1 | 0.144286 | 1.172556 |
| CBLB | 0.144478 | 1.17839 |
| RXRB | 0.14535 | 0.862069 |
| CMA1 | 0.145371 | 0.8255 |
| MAP2K1 | 0.145446 | 1.210153 |
| TAC1 | 0.146111 | 0.855276 |
| FGF11 | 0.146276 | 1.157169 |
| PPY | 0.148047 | 1.166772 |
| GALR3 | 0.148275 | 0.740137 |
| SLC29A3 | 0.15025 | 1.177332 |
| EBI3 | 0.150404 | 0.855899 |
| PSMD8 | 0.152139 | 1.202103 |
| HSPA1B | 0.152291 | 1.152424 |
| COLEC12 | 0.153521 | 1.1591 |
| BLNK | 0.153709 | 1.157208 |
| GNAI1 | 0.153809 | 0.86524 |
| CGB | 0.154461 | 1.145448 |
| TMSB15B | 0.154461 | 0.855392 |
| CALCB | 0.154728 | 0.835125 |
| NFKBIB | 0.155213 | 1.161138 |
| CALCR | 0.155865 | 0.856818 |
| ESRRA | 0.156146 | 1.14701 |
| CCR6 | 0.156228 | 0.833171 |
| GHRH | 0.156296 | 0.401483 |
| UCN | 0.157058 | 0.871475 |
| DEFA6 | 0.157199 | 0.827385 |
| CCL1 | 0.158706 | 1.153343 |
| CASP3 | 0.158843 | 1.185663 |
| ISG20L2 | 0.159466 | 1.175809 |
| BACH2 | 0.159745 | 0.847743 |
| ORM2 | 0.160388 | 1.135816 |
| TSHR | 0.160706 | 0.84112 |
| FABP3 | 0.160942 | 1.163368 |
| HTR3D | 0.162617 | 0.850331 |
| VEGFB | 0.162785 | 0.876423 |
| NOX5 | 0.163262 | 0.851398 |
| EPGN | 0.163486 | 1.098414 |
| GAL | 0.164353 | 1.148668 |
| INS | 0.164805 | 1.160616 |
| FGF19 | 0.165118 | 1.152773 |
| CYR61 | 0.166172 | 1.166947 |
| NPPC | 0.167122 | 0.814657 |
| IL12B | 0.167577 | 0.856557 |
| FGF7 | 0.168201 | 1.156801 |
| PRLH | 0.169158 | 0.847637 |
| SEMA4A | 0.169185 | 1.140386 |
| SHFM1 | 0.17036 | 1.169071 |
| PSMC2 | 0.170539 | 1.194188 |
| AZGP1 | 0.171071 | 1.15221 |
| HLA-DQB1 | 0.172446 | 1.143538 |
| IL2 | 0.172959 | 0.84523 |
| PSMC6 | 0.173542 | 1.189793 |
| CBL | 0.174471 | 1.171061 |
| MLN | 0.175092 | 0.825175 |
| KIR2DL4 | 0.175654 | 1.148401 |
| FAM19A5 | 0.176117 | 0.870733 |
| TAPBP | 0.177643 | 1.17261 |
| CXCL1 | 0.177739 | 1.143838 |
| PGLYRP1 | 0.178427 | 0.860173 |
| PGR | 0.178854 | 0.863638 |
| CD1D | 0.178972 | 0.868203 |
| NPY | 0.179213 | 0.861917 |
| IFNGR2 | 0.179701 | 1.143228 |
| BCL3 | 0.179811 | 1.150966 |
| SFTPA1 | 0.179849 | 1.122273 |
| UBR1 | 0.180368 | 1.165383 |
| CD86 | 0.180664 | 1.148738 |
| HRAS | 0.18135 | 0.874127 |
| CXCR3 | 0.181745 | 1.153221 |
| EED | 0.182307 | 1.159355 |
| FABP4 | 0.182565 | 0.862112 |
| SOCS1 | 0.18379 | 0.865641 |
| ADCYAP1R1 | 0.184589 | 0.862565 |
| FOS | 0.184701 | 1.156337 |
| PF4V1 | 0.185483 | 1.147938 |
| DEFB132 | 0.186448 | 0.732042 |
| CGB2 | 0.186576 | 1.133342 |
| EPO | 0.186949 | 1.139762 |
| KIR3DL3 | 0.187188 | 1.127367 |
| HSP90AA1 | 0.187704 | 1.15818 |
| SEMA6B | 0.189734 | 0.871578 |
| CXCL16 | 0.190314 | 1.147312 |
| RNASE3 | 0.190578 | 1.140395 |
| CREB1 | 0.193196 | 1.15627 |
| IL17RD | 0.19356 | 1.142101 |
| CRP | 0.193823 | 1.150838 |
| DEFB124 | 0.195967 | 0.856522 |
| FGF10 | 0.196949 | 1.146118 |
| AGTR2 | 0.197808 | 0.839345 |
| FCGRT | 0.197939 | 0.872303 |
| IFNA5 | 0.198083 | 0.852686 |
| HMOX1 | 0.198226 | 1.140005 |
| CD1A | 0.198268 | 1.14613 |
| PAEP | 0.200207 | 1.125411 |
| PSMC1 | 0.200237 | 1.180164 |
| CYSLTR2 | 0.200643 | 0.876481 |
| CALR | 0.20101 | 1.179113 |
| S100A7A | 0.201204 | 1.102152 |
| DAXX | 0.201508 | 1.158206 |
| IL17RC | 0.204048 | 0.881607 |
| DEFA5 | 0.205243 | 0.861152 |
| IL2RA | 0.205844 | 1.142147 |
| CX3CR1 | 0.206968 | 0.874808 |
| LTBP4 | 0.207457 | 0.873286 |
| CD22 | 0.208057 | 0.859457 |
| KIR2DL1 | 0.208232 | 0.850125 |
| APOBEC3A | 0.208486 | 1.147321 |
| PSMD4 | 0.210624 | 1.143525 |
| OBP2B | 0.21109 | 1.134514 |
| LTBP3 | 0.21215 | 0.885103 |
| QRFP | 0.213159 | 0.870304 |
| HCST | 0.214517 | 0.870208 |
| INHBC | 0.215258 | 0.869723 |
| SBDS | 0.215657 | 1.165904 |
| HDAC1 | 0.215849 | 1.142348 |
| CXCR5 | 0.216152 | 0.841415 |
| C20orf70 | 0.217708 | 0.799613 |
| JAG2 | 0.217874 | 0.886747 |
| MAPK3 | 0.218345 | 1.148305 |
| EDNRB | 0.218538 | 0.876113 |
| S100A7 | 0.219478 | 1.124077 |
| HTR1A | 0.221251 | 0.857816 |
| RFXANK | 0.225455 | 1.158201 |
| IRF7 | 0.225693 | 1.135104 |
| PSMC5 | 0.225965 | 0.883934 |
| CSF3 | 0.227757 | 0.860518 |
| MCHR2 | 0.227814 | 1.125681 |
| GNRHR | 0.22782 | 0.877656 |
| ACVR2B | 0.228527 | 0.883492 |
| WFDC2 | 0.229438 | 1.140674 |
| BMP10 | 0.229764 | 0.811938 |
| IL10RA | 0.230184 | 0.882085 |
| CRH | 0.230395 | 0.877812 |
| IFNE | 0.230937 | 1.126857 |
| ANGPTL6 | 0.231459 | 0.855122 |
| LEFTY1 | 0.231716 | 1.132484 |
| TRH | 0.232392 | 0.873945 |
| ESRRG | 0.233916 | 0.880715 |
| MAP3K8 | 0.235527 | 1.135485 |
| PTGER2 | 0.236018 | 1.127783 |
| RAET1L | 0.236415 | 1.119284 |
| NR1D1 | 0.23704 | 1.141003 |
| PRKCB | 0.238484 | 0.875885 |
| CD81 | 0.240145 | 0.904433 |
| DEFB126 | 0.243649 | 1.105428 |
| IL18RAP | 0.244141 | 0.877536 |
| TLR2 | 0.244186 | 1.120205 |
| ENG | 0.244395 | 0.889574 |
| CCR1 | 0.244575 | 1.126353 |
| LBP | 0.247077 | 0.875119 |
| NR1I3 | 0.248006 | 0.884471 |
| TNFRSF1B | 0.249099 | 1.121503 |
| CD79B | 0.249848 | 0.87289 |
| RXFP3 | 0.249924 | 0.852481 |
| IL1F5 | 0.251485 | 1.097541 |
| TNF | 0.251791 | 0.884494 |
| HLA-DMB | 0.252845 | 1.121089 |
| NR1H4 | 0.254249 | 1.128656 |
| PLXNB2 | 0.254615 | 1.125586 |
| TG | 0.254974 | 1.094188 |
| RNASEL | 0.255556 | 1.126042 |
| ZC3HAV1L | 0.256896 | 1.126485 |
| LYZ | 0.257771 | 1.117115 |
| AMBN | 0.257921 | 0.826302 |
| NCR3 | 0.258633 | 0.872102 |
| AKT3 | 0.259349 | 0.888589 |
| TPM2 | 0.259666 | 1.121141 |
| IFNA17 | 0.259813 | 0.856171 |
| DEFA4 | 0.260264 | 0.874628 |
| TPT1 | 0.260274 | 1.13297 |
| PLXNC1 | 0.260831 | 0.889135 |
| TNFRSF13B | 0.261169 | 0.86538 |
| HLA-G | 0.26183 | 1.125719 |
| IL15 | 0.262254 | 1.129397 |
| HSPA5 | 0.262834 | 1.12534 |
| PTK2 | 0.2634 | 1.128005 |
| S1PR1 | 0.263543 | 0.883065 |
| NRP1 | 0.264861 | 1.136584 |
| STC1 | 0.266225 | 1.129188 |
| FAM3D | 0.266717 | 1.116649 |
| GRB2 | 0.267455 | 0.890195 |
| LECT2 | 0.270794 | 0.780946 |
| ADAR | 0.270955 | 1.13454 |
| CALCA | 0.271104 | 0.86099 |
| ORM1 | 0.271688 | 1.103245 |
| CRLF3 | 0.273824 | 1.131355 |
| SOS1 | 0.274741 | 1.133749 |
| GDF15 | 0.275083 | 1.115691 |
| CCL27 | 0.275694 | 0.882793 |
| GCGR | 0.276006 | 0.889002 |
| TAPBPL | 0.277067 | 0.889414 |
| SLC10A2 | 0.27781 | 0.872551 |
| TRIM27 | 0.278548 | 0.892025 |
| PPBP | 0.279179 | 1.109011 |
| FGF20 | 0.279214 | 1.112103 |
| TIE1 | 0.279694 | 0.894416 |
| FPR2 | 0.281578 | 1.116461 |
| DEFB131 | 0.283993 | 0.85612 |
| ADIPOR2 | 0.284518 | 1.092666 |
| FABP7 | 0.285099 | 0.84812 |
| ARMET | 0.286096 | 1.112868 |
| LCN8 | 0.286715 | 0.846703 |
| ICAM2 | 0.28713 | 0.892589 |
| HSP90AB1 | 0.287773 | 1.130907 |
| SFTPD | 0.288482 | 0.894952 |
| NEO1 | 0.289243 | 0.887921 |
| CSH1 | 0.289716 | 1.087819 |
| NFKB1 | 0.289823 | 1.125622 |
| AKT2 | 0.294323 | 1.135667 |
| HTN1 | 0.295324 | 0.779071 |
| SEMA5B | 0.297941 | 0.891614 |
| SST | 0.298195 | 0.898021 |
| CXCL2 | 0.298442 | 1.10894 |
| TBK1 | 0.301643 | 1.132285 |
| ALB | 0.302709 | 1.118713 |
| LANCL1 | 0.303391 | 1.123988 |
| HSPA6 | 0.303894 | 1.101392 |
| VIM | 0.304034 | 1.127519 |
| KLRC4 | 0.305171 | 0.8907 |
| NFATC2 | 0.305301 | 0.891286 |
| LEP | 0.30787 | 0.881601 |
| PIK3R2 | 0.309026 | 0.891176 |
| TNFRSF13C | 0.311435 | 0.884595 |
| ELN | 0.312131 | 1.105673 |
| CYLD | 0.314222 | 0.898732 |
| F2R | 0.31461 | 1.115799 |
| IL1A | 0.315201 | 1.096823 |
| REG3G | 0.315891 | 1.110103 |
| NR5A1 | 0.317038 | 0.890912 |
| BMP7 | 0.319176 | 0.9014 |
| CCL2 | 0.320546 | 0.902845 |
| CTSG | 0.321102 | 0.893326 |
| BPIL3 | 0.321845 | 0.69054 |
| VIPR2 | 0.322856 | 0.888478 |
| HTR3A | 0.324662 | 1.097667 |
| ACO1 | 0.324663 | 1.104732 |
| CCL25 | 0.32913 | 0.903041 |
| DEFB125 | 0.329721 | 0.858813 |
| SERPINA3 | 0.332987 | 1.098785 |
| ERAP1 | 0.335009 | 1.117964 |
| IL22RA2 | 0.340546 | 1.106741 |
| HLA-DOA | 0.342039 | 1.102204 |
| NOS2 | 0.342599 | 1.097605 |
| STAT3 | 0.34354 | 1.120398 |
| HTN3 | 0.343668 | 0.628743 |
| PMP2 | 0.344278 | 0.898421 |
| VEGFA | 0.346872 | 1.102757 |
| C5AR1 | 0.349542 | 1.104998 |
| DCD | 0.349982 | 1.091176 |
| GIP | 0.351397 | 0.909162 |
| NFATC3 | 0.352113 | 1.102668 |
| BPIL2 | 0.353489 | 1.107274 |
| MC2R | 0.356311 | 1.111176 |
| HLA-DPA1 | 0.356811 | 1.097487 |
| NFKBIZ | 0.357156 | 1.098911 |
| ACVR1C | 0.358434 | 0.89894 |
| MLNR | 0.358808 | 0.878119 |
| IL22 | 0.359484 | 0.860991 |
| ARAF | 0.360105 | 0.913524 |
| KLRD1 | 0.363081 | 1.103176 |
| IFNA10 | 0.363274 | 0.881821 |
| OXT | 0.363573 | 0.895554 |
| FPR1 | 0.363985 | 1.095987 |
| C3AR1 | 0.364381 | 1.096049 |
| SKIV2L | 0.366315 | 0.911313 |
| IL28RA | 0.366701 | 0.90885 |
| PLXNA3 | 0.366914 | 0.912573 |
| UBXN1 | 0.369073 | 1.103477 |
| STC2 | 0.36972 | 0.913934 |
| INHBE | 0.369979 | 0.904237 |
| SCT | 0.37027 | 1.097222 |
| BMP8B | 0.373393 | 1.102531 |
| NR5A2 | 0.374164 | 1.095038 |
| NUDT6 | 0.3748 | 0.914033 |
| SOCS3 | 0.376651 | 1.095225 |
| TNFRSF10C | 0.378528 | 1.090139 |
| CCL21 | 0.380373 | 0.911552 |
| PTH2 | 0.384692 | 0.897055 |
| RETN | 0.384927 | 1.089419 |
| IL23R | 0.385254 | 0.909933 |
| FCN2 | 0.386713 | 0.896072 |
| FGF1 | 0.386945 | 0.919261 |
| HTR3B | 0.387319 | 0.901651 |
| IL17RA | 0.388322 | 1.096495 |
| PDGFB | 0.392293 | 1.10212 |
| CD247 | 0.392593 | 0.912256 |
| TLR4 | 0.394681 | 1.093493 |
| 7-Sep | 0.397397 | 1.108356 |
| IFNA1 | 0.397513 | 1.08191 |
| TNFAIP3 | 0.397519 | 1.093319 |
| SPINK5 | 0.397897 | 1.08618 |
| CCBP2 | 0.399186 | 0.904722 |
| JUN | 0.400049 | 1.094058 |
| CD1E | 0.400481 | 0.915167 |
| TGFB3 | 0.406359 | 1.087834 |
| AVPR1A | 0.406587 | 1.087165 |
| PGRMC2 | 0.407496 | 0.917459 |
| SEMG2 | 0.408579 | 1.11668 |
| NCR2 | 0.409658 | 0.904005 |
| CRLF1 | 0.410287 | 1.087238 |
| APOD | 0.41378 | 1.084875 |
| CYBB | 0.414194 | 1.084795 |
| MIF | 0.41546 | 0.923423 |
| FABP9 | 0.417195 | 0.91124 |
| NR2F6 | 0.417196 | 1.093857 |
| IL6 | 0.417858 | 1.085981 |
| SOS2 | 0.418137 | 0.917637 |
| NOX3 | 0.418621 | 1.081977 |
| PPARA | 0.419124 | 1.085573 |
| NOD1 | 0.420889 | 1.089651 |
| CANX | 0.421343 | 1.093298 |
| CLEC4M | 0.424041 | 0.766561 |
| GMFB | 0.425011 | 1.095114 |
| TDGF3 | 0.425507 | 1.088386 |
| CCL24 | 0.426008 | 1.077913 |
| IREB2 | 0.432261 | 1.094148 |
| TYROBP | 0.432425 | 1.085605 |
| HNF4A | 0.434402 | 0.924054 |
| GNLY | 0.435013 | 1.087837 |
| IL13RA1 | 0.435956 | 1.088632 |
| LGR5 | 0.436773 | 1.085446 |
| MASP1 | 0.437351 | 0.913014 |
| FCGR3B | 0.439616 | 1.078189 |
| HLA-A | 0.44041 | 1.093538 |
| IL1F10 | 0.440417 | 1.06828 |
| CXCR4 | 0.441282 | 1.079604 |
| NGF | 0.441796 | 0.920135 |
| IL18R1 | 0.442674 | 0.92473 |
| BRAF | 0.442807 | 0.921523 |
| APLNR | 0.443658 | 0.924911 |
| PGLYRP2 | 0.444412 | 0.921632 |
| FGF4 | 0.444519 | 1.068431 |
| TNFRSF19 | 0.445202 | 0.929152 |
| ESR1 | 0.446494 | 0.91838 |
| LCK | 0.448274 | 1.079798 |
| CD1B | 0.448298 | 0.915256 |
| ACVR1B | 0.449196 | 1.08655 |
| IL34 | 0.44945 | 0.92884 |
| PAK4 | 0.450304 | 1.087714 |
| NPPB | 0.451962 | 0.920549 |
| NCK2 | 0.453681 | 1.071629 |
| NODAL | 0.453837 | 1.083605 |
| IL24 | 0.453883 | 0.916292 |
| IL12RB1 | 0.453904 | 1.080078 |
| CSF2RA | 0.454585 | 1.078773 |
| CD40LG | 0.456284 | 0.920078 |
| HAMP | 0.458174 | 1.082228 |
| PLXNB3 | 0.458184 | 1.078567 |
| LGMN | 0.459743 | 1.080011 |
| AZU1 | 0.460122 | 0.920079 |
| VAV3 | 0.460661 | 0.927266 |
| DDX17 | 0.461376 | 0.926792 |
| TNFSF18 | 0.46222 | 0.93045 |
| IFNA2 | 0.462268 | 1.065191 |
| PDGFD | 0.463256 | 1.086773 |
| CCR3 | 0.463573 | 1.073422 |
| GDF6 | 0.46471 | 1.078228 |
| PTGDR | 0.466146 | 0.919588 |
| ULBP2 | 0.467928 | 1.077882 |
| GRP | 0.469072 | 1.075282 |
| IL17F | 0.471056 | 0.884498 |
| SDC2 | 0.472109 | 0.928032 |
| CD1C | 0.47259 | 0.925993 |
| IL1F8 | 0.472773 | 1.058281 |
| SH2D1B | 0.47367 | 1.08222 |
| RNASE7 | 0.47382 | 1.072301 |
| CD19 | 0.474648 | 0.918797 |
| LTA | 0.474745 | 0.922663 |
| AMELX | 0.475767 | 1.083399 |
| MC4R | 0.475849 | 0.917812 |
| FGF16 | 0.480311 | 1.077487 |
| IL31 | 0.480918 | 1.083962 |
| PRKCA | 0.481193 | 1.075759 |
| PTH | 0.481672 | 0.929366 |
| NRG3 | 0.482115 | 1.077041 |
| NTF3 | 0.482639 | 1.076235 |
| BPI | 0.483064 | 1.080244 |
| SPAG11B | 0.484659 | 0.907315 |
| C20orf114 | 0.48474 | 1.0758 |
| ITK | 0.485248 | 0.925062 |
| BMP8A | 0.486415 | 1.077516 |
| UTS2 | 0.487228 | 1.07063 |
| RORB | 0.487549 | 1.076012 |
| PNOC | 0.490766 | 0.92268 |
| C20orf185 | 0.490844 | 0.916416 |
| TEK | 0.491167 | 0.929314 |
| RARB | 0.494343 | 1.073749 |
| INSL4 | 0.495819 | 0.918247 |
| TCHHL1 | 0.496118 | 0.912446 |
| IKBKB | 0.497144 | 1.070372 |
| NLRX1 | 0.498572 | 1.077569 |
| TLR8 | 0.500223 | 1.068004 |
| MALT1 | 0.501998 | 1.073515 |
| DEFB105A | 0.504292 | 1.061227 |
| KIR3DL1 | 0.504668 | 0.934115 |
| IL5RA | 0.505496 | 0.916301 |
| RXFP1 | 0.50574 | 0.926405 |
| SAA1 | 0.506649 | 1.063604 |
| TXK | 0.507397 | 0.92658 |
| CSHL1 | 0.508863 | 1.074542 |
| IL16 | 0.511896 | 0.934419 |
| TNC | 0.514634 | 1.072469 |
| PMCH | 0.517337 | 1.07091 |
| BPHL | 0.517859 | 0.936144 |
| GPHB5 | 0.518129 | 1.069075 |
| CMTM2 | 0.51859 | 1.063952 |
| FASLG | 0.520232 | 0.93416 |
| C19orf10 | 0.523011 | 1.068734 |
| PTH2R | 0.525068 | 1.06537 |
| ANGPT1 | 0.525731 | 1.066307 |
| NDP | 0.526536 | 1.067536 |
| TDGF1 | 0.527189 | 0.927397 |
| ILK | 0.533807 | 1.077463 |
| C20orf71 | 0.533907 | 0.532338 |
| ABCC4 | 0.535992 | 0.941058 |
| TNFRSF14 | 0.537205 | 1.065663 |
| IL12A | 0.537968 | 1.063913 |
| CCL11 | 0.53934 | 1.062399 |
| P11 | 0.541114 | 1.05628 |
| CSF1 | 0.543492 | 1.064294 |
| SH2D1A | 0.544019 | 0.936688 |
| TGFB1 | 0.544092 | 1.068472 |
| AR | 0.545292 | 1.066638 |
| DEFB108B | 0.549992 | 0.930688 |
| DUOX1 | 0.55037 | 1.061603 |
| HSPA4 | 0.551461 | 1.067906 |
| ANGPTL1 | 0.554733 | 0.939598 |
| PTK2B | 0.555132 | 0.942284 |
| SEMG1 | 0.557898 | 1.061888 |
| NR2F2 | 0.558525 | 1.062127 |
| TSLP | 0.559081 | 1.058748 |
| NR2C2 | 0.560091 | 0.940304 |
| SCYE1 | 0.560278 | 1.067735 |
| CD72 | 0.56069 | 1.063567 |
| HLA-DPB1 | 0.562988 | 1.059321 |
| CSH2 | 0.56789 | 1.038635 |
| LACRT | 0.568091 | 0.651322 |
| CCL19 | 0.569096 | 0.942858 |
| ACVRL1 | 0.57011 | 0.942318 |
| CD28 | 0.57328 | 0.93984 |
| ROBO1 | 0.574426 | 1.061979 |
| DEFA3 | 0.574812 | 1.055754 |
| SAA2 | 0.577394 | 1.053482 |
| DCK | 0.57757 | 0.941727 |
| TNFRSF25 | 0.578351 | 0.945428 |
| HLA-DOB | 0.583673 | 1.05746 |
| TFR2 | 0.585617 | 0.946411 |
| TUBB3 | 0.585748 | 1.059407 |
| PTH1R | 0.58977 | 0.943843 |
| KGFLP1 | 0.595097 | 1.057777 |
| PYY | 0.597633 | 1.052682 |
| IFNA6 | 0.598986 | 1.063844 |
| GPHA2 | 0.602898 | 1.057965 |
| FGA | 0.603057 | 0.95008 |
| VAV1 | 0.603247 | 1.053384 |
| PPP3R2 | 0.603889 | 1.062895 |
| PIK3R1 | 0.605028 | 0.946644 |
| KIAA0368 | 0.605555 | 0.947067 |
| TNFRSF17 | 0.606977 | 0.948542 |
| NOX1 | 0.607774 | 1.055663 |
| AP3B1 | 0.610027 | 1.061519 |
| SLC40A1 | 0.610524 | 1.05448 |
| THPO | 0.610617 | 0.951949 |
| ITGAL | 0.617202 | 0.950469 |
| DAK | 0.617309 | 1.050347 |
| IL1RL2 | 0.617856 | 0.945645 |
| APLN | 0.61868 | 0.949775 |
| FABP2 | 0.619101 | 0.951742 |
| TMSB4Y | 0.620469 | 0.947867 |
| CTGF | 0.623839 | 1.054391 |
| SORT1 | 0.624136 | 0.949793 |
| PIK3R5 | 0.624621 | 0.950898 |
| IL26 | 0.62539 | 0.943419 |
| FGFR4 | 0.627218 | 0.953962 |
| FGF6 | 0.627696 | 0.925997 |
| SEMA3D | 0.628121 | 0.948856 |
| BTC | 0.628523 | 0.950801 |
| KIR2DL3 | 0.629258 | 1.04746 |
| GALR2 | 0.629384 | 0.934304 |
| ZC3HAV1 | 0.630957 | 1.056579 |
| GFAP | 0.634898 | 0.948353 |
| SCGB3A1 | 0.636825 | 0.948739 |
| RETNLB | 0.639332 | 0.947015 |
| GDNF | 0.640421 | 1.050941 |
| DEFB136 | 0.643244 | 1.052522 |
| OPRK1 | 0.645477 | 0.953914 |
| PLUNC | 0.645501 | 0.947218 |
| MBL2 | 0.646099 | 0.95569 |
| PLA2G2A | 0.64642 | 0.952613 |
| OBP2A | 0.646816 | 1.050801 |
| CMTM4 | 0.648342 | 0.951854 |
| GLP2R | 0.649008 | 0.954762 |
| SEMA4C | 0.649024 | 1.050363 |
| GNRH1 | 0.649077 | 0.954662 |
| PLXNA4 | 0.652881 | 0.95417 |
| IFNA8 | 0.654358 | 1.048269 |
| PSMD3 | 0.655684 | 1.052005 |
| CCR4 | 0.656431 | 0.953316 |
| CTF1 | 0.661737 | 1.044904 |
| GDF3 | 0.663332 | 0.953641 |
| MCHR1 | 0.666235 | 1.043964 |
| ACTA1 | 0.667122 | 0.949213 |
| NFYC | 0.667506 | 1.04624 |
| GRN | 0.668799 | 0.954942 |
| GREM2 | 0.669313 | 0.954769 |
| AGRP | 0.669517 | 1.046679 |
| NR3C1 | 0.671832 | 1.046791 |
| HTR3E | 0.672441 | 0.949472 |
| PTGFR | 0.672599 | 0.953903 |
| IL33 | 0.673249 | 0.956231 |
| BMP3 | 0.673323 | 1.046982 |
| LEFTY2 | 0.673423 | 0.953925 |
| NR2F1 | 0.675287 | 0.958934 |
| LILRB3 | 0.677422 | 0.956315 |
| VIP | 0.677582 | 0.956492 |
| RAET1G | 0.677969 | 1.046915 |
| IL17C | 0.679182 | 0.956012 |
| FGF5 | 0.680244 | 1.038278 |
| CIITA | 0.682821 | 1.041642 |
| FGF3 | 0.68393 | 1.04445 |
| NR6A1 | 0.685678 | 0.961713 |
| PRKCQ | 0.686719 | 0.958281 |
| PPP3CC | 0.686731 | 0.962083 |
| HTR3C | 0.688075 | 1.04584 |
| GHSR | 0.68913 | 1.043158 |
| CD70 | 0.689836 | 1.040336 |
| CD48 | 0.690152 | 0.95902 |
| TMSB15A | 0.691169 | 1.041426 |
| IL9 | 0.691428 | 1.028726 |
| LALBA | 0.692731 | 0.954519 |
| BMP5 | 0.69381 | 0.959014 |
| NR2C1 | 0.693879 | 0.961235 |
| IL10 | 0.695294 | 0.957881 |
| LTF | 0.695514 | 1.041451 |
| NFKBIA | 0.697138 | 1.044399 |
| IL8RA | 0.697923 | 1.038206 |
| KLRC1 | 0.700371 | 1.041642 |
| SPINLW1 | 0.701218 | 1.044998 |
| NFATC4 | 0.702077 | 0.960769 |
| FLT3 | 0.705676 | 0.96225 |
| CD244 | 0.707685 | 0.961961 |
| IL19 | 0.711186 | 1.031252 |
| CCL8 | 0.712876 | 0.960519 |
| XCL1 | 0.714724 | 1.039637 |
| IL25 | 0.715281 | 0.962477 |
| APOBEC3H | 0.715612 | 1.037863 |
| TNFRSF9 | 0.71594 | 1.037838 |
| LCN9 | 0.716644 | 0.937214 |
| CHUK | 0.717024 | 1.038668 |
| OPRM1 | 0.717439 | 0.936603 |
| TNFSF15 | 0.719306 | 1.035703 |
| ROBO3 | 0.719736 | 1.03699 |
| LCP2 | 0.720261 | 1.036848 |
| IL5 | 0.723411 | 0.96089 |
| SYTL1 | 0.724947 | 1.036545 |
| NR0B2 | 0.726839 | 0.963192 |
| SHC4 | 0.726959 | 0.962576 |
| IL12RB2 | 0.72824 | 1.034624 |
| TNFSF14 | 0.728479 | 0.96602 |
| IL1RL1 | 0.729869 | 0.962326 |
| OXTR | 0.730298 | 1.03671 |
| LMBR1 | 0.733817 | 0.966018 |
| DES | 0.734117 | 0.964733 |
| CR2 | 0.734889 | 0.962284 |
| RORC | 0.73562 | 0.964902 |
| AVP | 0.737886 | 1.039821 |
| IFNK | 0.739585 | 1.034967 |
| NFYB | 0.740048 | 0.963912 |
| PPBPL2 | 0.741243 | 1.032268 |
| CHIT1 | 0.745016 | 1.034816 |
| SDC3 | 0.746386 | 0.9678 |
| CCL26 | 0.746923 | 0.968812 |
| RARA | 0.749599 | 1.033941 |
| CCL17 | 0.750695 | 1.034412 |
| ARRB1 | 0.750698 | 1.03417 |
| ULBP1 | 0.755188 | 1.031763 |
| PTGER1 | 0.756653 | 0.965906 |
| GPI | 0.756821 | 0.967263 |
| APOM | 0.757368 | 0.966938 |
| KIR3DL2 | 0.757684 | 0.968664 |
| IGF2R | 0.75845 | 1.034839 |
| DMBT1 | 0.758558 | 1.031617 |
| LCN1 | 0.760841 | 0.974476 |
| CAMP | 0.762357 | 1.03383 |
| CSRP1 | 0.765184 | 1.031739 |
| ANGPTL7 | 0.767265 | 0.968643 |
| PRL | 0.768298 | 0.968573 |
| CMKLR1 | 0.769623 | 0.970881 |
| CCR9 | 0.770496 | 0.970306 |
| IFI30 | 0.770662 | 1.03106 |
| NR4A1 | 0.770977 | 1.033785 |
| CD209 | 0.771322 | 0.967267 |
| IRF9 | 0.77189 | 1.030666 |
| HSPA8 | 0.773107 | 1.033046 |
| DEFB137 | 0.773608 | 1.03193 |
| PLCG2 | 0.77375 | 0.971697 |
| GH2 | 0.773939 | 1.027888 |
| IL7R | 0.774298 | 1.030259 |
| CD3G | 0.775671 | 0.970821 |
| CD79A | 0.775775 | 0.970088 |
| GCG | 0.776429 | 1.028703 |
| PSMD10 | 0.777935 | 0.970376 |
| IL9R | 0.779101 | 0.971744 |
| CCR8 | 0.779433 | 0.970961 |
| IL17A | 0.779944 | 0.966741 |
| PRF1 | 0.781515 | 1.030069 |
| A2M | 0.784252 | 0.972994 |
| BMPR1A | 0.784291 | 1.030138 |
| IGF1 | 0.786642 | 0.970531 |
| IL20 | 0.788822 | 1.028435 |
| NOV | 0.793773 | 1.028579 |
| FGF21 | 0.79705 | 1.026719 |
| PENK | 0.80051 | 0.974394 |
| NR4A3 | 0.803075 | 0.973602 |
| PTPN11 | 0.803387 | 1.028181 |
| ROBO2 | 0.803631 | 0.973655 |
| KIR2DS4 | 0.804013 | 1.025972 |
| ARTN | 0.806104 | 1.026632 |
| VAV2 | 0.806631 | 1.022522 |
| FSHR | 0.808798 | 0.975726 |
| FABP5 | 0.809617 | 1.023769 |
| PIK3CG | 0.809674 | 1.023979 |
| LCNL1 | 0.810697 | 0.972781 |
| AEN | 0.812553 | 0.975282 |
| LEPR | 0.814565 | 0.976131 |
| NOS1 | 0.814977 | 0.977735 |
| COLEC10 | 0.815369 | 0.979094 |
| NR1H2 | 0.816479 | 1.024752 |
| CSF2RB | 0.816898 | 0.976781 |
| IL3 | 0.820019 | 1.028548 |
| CD4 | 0.821756 | 1.022952 |
| CDK4 | 0.823146 | 1.028797 |
| CALCRL | 0.827057 | 0.977151 |
| S100G | 0.829695 | 1.019897 |
| LHCGR | 0.830044 | 0.977307 |
| THRB | 0.830308 | 1.023606 |
| IRF3 | 0.837035 | 0.979022 |
| S100A7L2 | 0.837297 | 0.975086 |
| R3HDML | 0.839242 | 1.021385 |
| CXCL12 | 0.840101 | 0.978419 |
| IL27RA | 0.84292 | 1.019983 |
| CTSL1 | 0.845945 | 1.02099 |
| INHA | 0.846112 | 0.979833 |
| PDYN | 0.85177 | 1.019233 |
| APOBEC3F | 0.851917 | 0.979735 |
| ACVR2A | 0.852715 | 0.979919 |
| HRG | 0.854442 | 1.018104 |
| SOD1 | 0.854839 | 0.980821 |
| ADRBK1 | 0.855236 | 1.019395 |
| VEGFC | 0.855303 | 0.981301 |
| CRABP1 | 0.855323 | 0.980604 |
| IL21R | 0.855669 | 1.019377 |
| JAK2 | 0.855862 | 1.019716 |
| SHC3 | 0.859044 | 1.01869 |
| ICOS | 0.861889 | 0.981852 |
| DEFB119 | 0.862521 | 0.978789 |
| CCL22 | 0.862799 | 0.982505 |
| CXCL13 | 0.865307 | 0.981908 |
| BECN1 | 0.867891 | 0.980372 |
| RORA | 0.868253 | 0.981771 |
| RASGRP1 | 0.868542 | 1.016703 |
| PGF | 0.868637 | 0.98265 |
| RBP4 | 0.871895 | 1.016487 |
| HFE2 | 0.872715 | 0.98342 |
| IL8RB | 0.874293 | 1.015619 |
| CELA1 | 0.874873 | 0.979619 |
| NR1D2 | 0.875772 | 0.983914 |
| RXFP2 | 0.875871 | 1.015897 |
| RBP2 | 0.878534 | 0.985185 |
| SEMA3E | 0.879293 | 0.983172 |
| HCK | 0.880868 | 1.015165 |
| ESM1 | 0.8824 | 1.01535 |
| NCR1 | 0.882705 | 1.015119 |
| NDRG1 | 0.883856 | 1.014701 |
| CD3D | 0.884353 | 1.014828 |
| IL1F9 | 0.887231 | 1.014367 |
| OGN | 0.887398 | 0.985581 |
| PLXND1 | 0.889626 | 1.01534 |
| IFNA13 | 0.890395 | 0.985073 |
| GLP1R | 0.891576 | 0.986773 |
| SPAG11A | 0.89185 | 0.98637 |
| RFX5 | 0.89188 | 1.014385 |
| IFNB1 | 0.891945 | 1.015559 |
| GMFG | 0.8925 | 0.985969 |
| FURIN | 0.893816 | 1.016619 |
| PTGER3 | 0.895189 | 1.014287 |
| PLXNA2 | 0.895202 | 1.014053 |
| PDGFRL | 0.897054 | 1.014562 |
| BTK | 0.897245 | 0.986797 |
| ADRM1 | 0.897707 | 0.986273 |
| AMHR2 | 0.90094 | 1.014231 |
| TNFSF8 | 0.903445 | 0.986974 |
| CXCR6 | 0.903608 | 1.012762 |
| INPP5D | 0.903988 | 0.988183 |
| PTPRC | 0.909111 | 1.011714 |
| PIK3CD | 0.912047 | 0.98853 |
| IFNA14 | 0.912752 | 0.988148 |
| PDCD1 | 0.91325 | 0.9883 |
| HSPA2 | 0.914824 | 0.98972 |
| AVPR2 | 0.916236 | 0.987425 |
| IRF5 | 0.917407 | 1.011325 |
| PTN | 0.918669 | 1.010389 |
| IL6ST | 0.918906 | 0.988863 |
| CYSLTR1 | 0.923684 | 1.011221 |
| XCL2 | 0.923808 | 0.989757 |
| MAPK8 | 0.925108 | 1.010724 |
| TRHR | 0.925528 | 1.008101 |
| SCTR | 0.926905 | 0.990836 |
| DEFB110 | 0.92763 | 1.006902 |
| FGFR3 | 0.93009 | 0.991636 |
| CTLA4 | 0.931142 | 1.009019 |
| TRPC4AP | 0.934058 | 0.990528 |
| GDF5 | 0.935908 | 1.009526 |
| LTB | 0.936904 | 1.007855 |
| S100A12 | 0.938128 | 1.008792 |
| IL2RB | 0.94235 | 0.992625 |
| CHP2 | 0.943126 | 0.99309 |
| INSL3 | 0.94427 | 1.006738 |
| ELAVL1 | 0.944497 | 1.007774 |
| GPR32 | 0.944749 | 1.007907 |
| HGF | 0.944888 | 1.00695 |
| SLIT2 | 0.94559 | 0.992927 |
| GUCA2A | 0.947304 | 1.006668 |
| FGF18 | 0.9482 | 0.993621 |
| TNFSF12 | 0.948766 | 1.006429 |
| CD3E | 0.948906 | 0.993479 |
| IL21 | 0.952193 | 1.006284 |
| IGF1R | 0.952553 | 0.994081 |
| CCR5 | 0.952787 | 1.00596 |
| CSK | 0.954546 | 0.993981 |
| PTX3 | 0.955074 | 0.994125 |
| TNFRSF18 | 0.955297 | 1.005978 |
| RASGRP3 | 0.956352 | 0.994339 |
| INSR | 0.957624 | 0.994961 |
| CLEC11A | 0.958476 | 1.005745 |
| TLR7 | 0.958959 | 0.994976 |
| PTGER4 | 0.960003 | 1.004844 |
| IL1B | 0.96029 | 1.005016 |
| CD8B | 0.960858 | 0.994797 |
| GALP | 0.961377 | 0.992765 |
| IAPP | 0.962847 | 0.995302 |
| AKT1 | 0.967356 | 0.995468 |
| CSF3R | 0.976131 | 0.997085 |
| PTGDS | 0.977709 | 1.002865 |
| CD14 | 0.979748 | 0.997318 |
| TGFBR3 | 0.981282 | 1.002391 |
| PDGFA | 0.982625 | 0.997857 |
| CSF1R | 0.985185 | 0.998136 |
| TNFRSF8 | 0.98558 | 1.001943 |
| DEFB4 | 0.986882 | 1.001903 |
| PDF | 0.988025 | 0.998401 |
| KNG1 | 0.989589 | 1.001323 |
| INSL6 | 0.990233 | 1.001168 |
| MTNR1A | 0.991513 | 1.001122 |
| CD8A | 0.992394 | 0.998994 |
| FLT1 | 0.993026 | 1.000894 |
| IFNA7 | 0.99421 | 0.162947 |
| DEFB105B | 0.995065 | 0.323488 |
| DEFB129 | 0.995131 | 0.323493 |
| FABP6 | 0.995306 | 0.999385 |
| IFNA4 | 0.995521 | 0.007601 |
| FGR | 0.995552 | 0.999433 |
| DEFB133 | 0.995562 | 0.205866 |
| UTS2D | 0.995578 | 0.999412 |
| DEFB121 | 0.995594 | 0.041406 |
| DEFB128 | 0.996205 | 0.299974 |
| CCK | 0.996933 | 0.999589 |
| IL17RB | 0.997051 | 0.999661 |
| MPO | 0.997219 | 1.000405 |
