# Supplementary material for: Immunity-Related Gene Signature Identifies Subtypes Benefitting From Adjuvant Chemotherapy or Potentially Responding to PD1/PD-L1 Blockage in Pancreatic Cancer
Source: Front Cell Dev Biol. 2021 Jun 23;9:682261. doi: 10.3389/fcell.2021.682261 (PMC8264789; doi:10.3389/fcell.2021.682261)
Supplement: Supplementary Table 2 — The risk score formula of immune-related 18-gene signature. [file Table_2.DOCX]

Supplemental Table 2. The risk score formula of immune-related 18-gene signature

| Gene symbol | Formula |
| --- | --- |
| BIRC5 | 0.311881637640432 × Z-score transformed -∆CT of BIRC5 + 1.09158573174151 |
| CKLF | 0.227127890127546 × Z-score transformed -∆CT of CKLF + 1.13563945063773 |
| CRABP2 | 0.175210239229446 × Z-score transformed -∆CT of CRABP2 + 0.613235837303063 |
| CXCL11 | 0.357432120520961 × Z-score transformed -∆CT of CXCL11 + 0.714864241041922 |
| DKK1 | 0.015508526770984 × Z-score transformed -∆CT of DKK1 + 0.031017053541968 |
| EREG | 0.106722463090457 × Z-score transformed -∆CT of EREG + 0.160083694635686 |
| FAM3C | 0.303287091158582 × Z-score transformed -∆CT of FAM3C + 1.21314836463433 |
| FGFRL1 | 0.067221552777096 × Z-score transformed -∆CT of FGFRL1 + 0.33610776388548 |
| FIGNL2 | (-0.833333333333334) × Z-score transformed -∆CT of FIGNL2 + 9.16666666666667 |
| GBP2 | 0.272784927119591 × Z-score transformed -∆CT of GBP2 + 1.22753217203816 |
| GDF9 | (-0.076443339593267) × Z-score transformed -∆CT of GDF9 + 0.458660037559601 |
| IL32 | (-0.067021341807543) × Z-score transformed -∆CT of IL32 + 0.100532012711314 |
| PSMB8 | (-0.127930027905461) × Z-score transformed -∆CT of PSMB8 + 0.319825069763654 |
| PSPN | (-0.438050303128155) × Z-score transformed -∆CT of PSPN + 1.9712263640767 |
| RFXAP | (-0.433944218136263) × Z-score transformed -∆CT of RFXAP + 1.51880476347692 |
| S100A11 | (-0.197588938245729) × Z-score transformed -∆CT of S100A11 + 0.395177876491458 |
| SDC4 | (-0.363266662147879) × Z-score transformed -∆CT of SDC4 + 0.726533324295758 |
| SLC22A17 | (-0.012339915012537) × Z-score transformed -∆CT of SLC22A17 + 0.061699575062685 |
