# Supplementary material for: Immunity-Related Gene Signature Identifies Subtypes Benefitting From Adjuvant Chemotherapy or Potentially Responding to PD1/PD-L1 Blockage in Pancreatic Cancer
Source: Front Cell Dev Biol. 2021 Jun 23;9:682261. doi: 10.3389/fcell.2021.682261 (PMC8264789; doi:10.3389/fcell.2021.682261)
Supplement: Supplementary Table 3 — Sequences of the qRT-PCR primers used in this study. [file Table_3.DOCX]

Supplemental Table 3. Sequences of the qRT-PCR primers used in this study.

| Name | Sequence | |
| --- | --- | --- |
|  | Forward | Reverse |
| BIRC5 | CCGCATCTCTACATTCAAGAAC | CTCCTTGAAGCAGAAGAAACAC |
| CKLF | TCGCAGAACCTACTCAGGCA | CAGCATCTTCACGTGGCCTT |
| CRABP2 | TGAATGTGATGCTGAGGAAGAT | TGGTGGAGGTTTTGATGTAGAA |
| CXCL11 | GCTGTGATATTGTGTGCTACAG | TTGGGTACATTATGGAGGCTTT |
| DKK1 | TACCAGACCATTGACAACTACC | TCCATTTTTGCAGTAATTCCCG |
| EREG | TGTGGCTTTGACCGTGATT | TCCCTGCCCATAAGTTTGA |
| FAM3C | GTTCTACAAAGCCTCCCAGATA | CTGCTCCACTTGCCATTTTAAA |
| FGFRL1 | GGCAAGGTCCACCAGCACATC | TGCAGCTCCGTCCTCCATCC |
| FIGNL2 | GTCGCCAACGCTGCCACTAC | AGGAAGGAGGCGTCGCTGTAC |
| GBP2 | CTTTAATGATCCTCGGTTGTGC | CTCTAGGTGAGCAAGGTACTTC |
| GDF9 | AGCTTCCTTCAATCTGAGTGAA | CCTTTACAGTATCGAGGGTTGT |
| IL32 | GCTTTTGTGACAAGGTCATGAG | ACTGGAAAGAGGACATGAAGAG |
| PSMB8 | CTTTAGATGACACGACCCTACC | CAATCTGAACGTTCCTTTCTCC |
| PSPN | CAATGGCCGTAGGGAAGTT | TTGCCACCTGTTCAGACGA |
| RFXAP | CTCAATTCACCCTGCTAGTGAT | CTCTAGATCCTCCAAACTAGCC |
| S100A11 | TCTCTCCAAGACAGAGTTCCTA | AGGCCACCAATCAGATTAAGAA |
| SDC4 | CTTGGTGCCTCTAGATAACCAT | GACACATCCTCACTCTCTTCAA |
| SLC22A17 | GCCTCAAGGATTGGGACTATAA | AAAGCCCAAGATGAAGAGGATC |
| GAPDH | GGAAGGTGAAGGTCGGAGTC | GTTGAGGTCAATGAAGGGGTC |
| PDL1 | GCTGCACTAATTGTCTATTGGG | CACAGTAATTCGCTTGTAGTCG |
