# Supplementary material for: Immunity-Related Gene Signature Identifies Subtypes Benefitting From Adjuvant Chemotherapy or Potentially Responding to PD1/PD-L1 Blockage in Pancreatic Cancer
Source: Front Cell Dev Biol. 2021 Jun 23;9:682261. doi: 10.3389/fcell.2021.682261 (PMC8264789; doi:10.3389/fcell.2021.682261)
Supplement: Supplementary Table 4 — The DEGs between the gemcitabine resistance population and main tumor cell population in GSE36563 dataset. [file Table_4.DOCX]

Supplemental Table 4. The DEGs between the gemcitabine resistance population and main tumor cell population in GSE 36563 dataset.

| Gene name | logFC | P.Value |
| --- | --- | --- |
| KCNC1 | 4.724748 | 4.46E-05 |
| FAM184A | 4.512156 | 9.76E-05 |
| CITED4 | 4.46384 | 8.47E-05 |
| PRKCQ | 4.428805 | 5.13E-05 |
| DPYSL5 | 4.297555 | 0.000101 |
| DSCAM | 3.977122 | 0.000161 |
| SAMD3 | 3.928316 | 0.000624 |
| NEFH | 3.721783 | 0.042452 |
| PACSIN1 | 3.638004 | 4.20E-05 |
| KCNJ8 | 3.505141 | 0.001477 |
| PHYHIPL | 3.232536 | 0.003423 |
| KCNH2 | 3.068128 | 0.001701 |
| MEOX2 | 3.043262 | 0.036844 |
| TBX21 | 2.980761 | 0.036196 |
| SEMA6D | 2.8548 | 0.003788 |
| C1QL3 | 2.82891 | 0.003822 |
| ACAP1 | 2.757884 | 0.016727 |
| GPR18 | 2.685297 | 0.006343 |
| PPM1J | 2.683338 | 0.000622 |
| GDNF | 2.673966 | 0.0188 |
| ELTD1 | 2.636595 | 0.001784 |
| ZBTB10 | 2.609456 | 0.000967 |
| PHACTR3 | 2.537921 | 0.041616 |
| SOX5 | 2.513347 | 0.035764 |
| PKNOX2 | 2.475439 | 0.01991 |
| IRF2BP1 | 2.47528 | 0.000538 |
| PGLYRP1 | 2.427319 | 3.07E-05 |
| D4S234E | 2.361624 | 0.005034 |
| DBX2 | 2.361009 | 0.024703 |
| HAS1 | 2.349409 | 0.014026 |
| FGF10 | 2.340019 | 0.040774 |
| ADAMTS9 | 2.33753 | 0.027874 |
| GJC1 | 2.33655 | 0.01838 |
| KLF12 | 2.328078 | 0.001758 |
| ZFPM2 | 2.305733 | 0.017853 |
| TAC1 | 2.290585 | 0.023339 |
| CPXM1 | 2.276788 | 0.046226 |
| FLNC | 2.254677 | 0.039634 |
| SEMA6A | 2.252635 | 0.002297 |
| TIE1 | 2.249593 | 0.011136 |
| MPDZ | 2.215523 | 0.042561 |
| ITGB3 | 2.207923 | 0.001193 |
| BACH2 | 2.197067 | 0.004503 |
| UNC45B | 2.18519 | 0.018034 |
| ARL4D | 2.181268 | 0.004989 |
| PGLYRP2 | 2.169436 | 0.015862 |
| SHANK3 | 2.159789 | 0.001223 |
| DTNA | 2.14147 | 0.005632 |
| CCRN4L | 2.128592 | 0.021126 |
| ELMOD1 | 2.114273 | 0.00057 |
| SIX6 | 2.110648 | 0.026284 |
| SRGAP3 | 2.107105 | 0.003097 |
| CERS4 | 2.106314 | 0.000768 |
| ARNTL | 2.100144 | 0.004466 |
| NRN1 | 2.096517 | 0.007764 |
| SORBS1 | 2.086882 | 0.009557 |
| GNAZ | 2.085122 | 0.007109 |
| F7 | 2.077 | 0.003045 |
| UBXN11 | 2.063027 | 0.00088 |
| ATP1A2 | 2.05339 | 0.003855 |
| GPM6A | 2.02189 | 0.009509 |
| FBLN7 | 2.017466 | 0.019406 |
| CHSY3 | 2.010522 | 0.030585 |
| GFRA1 | 1.990331 | 0.031658 |
| TNKS1BP1 | 1.983197 | 0.006568 |
| JAM2 | 1.958736 | 0.011321 |
| GPR88 | 1.929256 | 0.044391 |
| MEX3B | 1.923495 | 0.022817 |
| SOBP | 1.920648 | 0.025004 |
| ROBO4 | 1.912399 | 0.034021 |
| NKRF | 1.90711 | 0.001479 |
| CRYAB | 1.890768 | 0.041678 |
| LRFN5 | 1.881542 | 0.020471 |
| TECTB | 1.87942 | 0.006313 |
| KLHL29 | 1.878431 | 0.029163 |
| ABCA9 | 1.877134 | 0.027816 |
| ABCG2 | 1.861968 | 0.00723 |
| PDE7A | 1.839419 | 0.01665 |
| CDK17 | 1.828023 | 0.002247 |
| LOC100130507 | 1.827374 | 0.006615 |
| DGKA | 1.814748 | 0.005688 |
| PLEKHA2 | 1.812718 | 0.017081 |
| CHD5 | 1.810191 | 0.009273 |
| LEF1 | 1.781097 | 0.034991 |
| MARK1 | 1.776652 | 0.030724 |
| VTN | 1.775697 | 0.039031 |
| TSC22D2 | 1.77159 | 0.020759 |
| RFC1 | 1.768987 | 0.011457 |
| REV3L | 1.765159 | 0.004056 |
| LOC727982 | 1.755617 | 0.012571 |
| DNAJB4 | 1.748644 | 0.028979 |
| HTR5A | 1.748299 | 0.006768 |
| ZBTB41 | 1.742827 | 0.004538 |
| BPHL | 1.740326 | 0.004681 |
| TH | 1.713972 | 0.041941 |
| CPNE8 | 1.713747 | 0.045737 |
| TLE6 | 1.710982 | 0.038793 |
| GRAMD1A | 1.708342 | 0.009257 |
| SERPINC1 | 1.706167 | 0.026755 |
| SVEP1 | 1.704952 | 0.046131 |
| BMP15 | 1.704329 | 0.023666 |
| GAGE3 | 1.699097 | 0.022141 |
| ETS1 | 1.68953 | 0.008876 |
| ZNF280D | 1.685235 | 0.005618 |
| NAV3 | 1.68124 | 0.040585 |
| DMC1 | 1.677845 | 0.030646 |
| MED13 | 1.671697 | 0.009025 |
| ZNF625 | 1.668743 | 0.038518 |
| KCNQ4 | 1.668238 | 0.036574 |
| SIGLEC12 | 1.663742 | 0.005635 |
| CHD1 | 1.650664 | 0.007389 |
| STIM2 | 1.643498 | 0.002762 |
| MLLT4 | 1.633324 | 0.000711 |
| LMO1 | 1.63075 | 0.014672 |
| MLLT3 | 1.630606 | 0.015535 |
| DCD | 1.630194 | 0.028196 |
| POLR2K | 1.626529 | 0.011777 |
| SFT2D2 | 1.620553 | 0.030439 |
| UBR1 | 1.618416 | 0.002312 |
| SMC6 | 1.617118 | 0.009075 |
| ADRA2B | 1.616703 | 0.004505 |
| RNF146 | 1.60718 | 0.003794 |
| LILRA4 | 1.605267 | 0.034505 |
| PRKCA | 1.603757 | 0.006076 |
| ZBTB33 | 1.601265 | 0.017611 |
| RNF144A | 1.599031 | 0.024445 |
| EPHA10 | 1.597114 | 0.013649 |
| BCL6 | 1.594608 | 0.003276 |
| ZMYND17 | 1.585327 | 0.04546 |
| SMTN | 1.58343 | 0.043109 |
| ANKRD28 | 1.582929 | 0.016991 |
| PLK4 | 1.575384 | 0.032259 |
| NMD3 | 1.575084 | 0.030795 |
| ZBTB16 | 1.572449 | 0.041003 |
| AHDC1 | 1.5687 | 0.019518 |
| NELF | 1.563567 | 0.004327 |
| BAI2 | 1.562525 | 0.015316 |
| BRIX1 | 1.554235 | 0.003121 |
| RSF1 | 1.553177 | 0.009922 |
| TAF1L | 1.54814 | 0.012211 |
| FPR3 | 1.546045 | 0.037553 |
| EML2 | 1.544556 | 0.004928 |
| SGCD | 1.536361 | 0.03075 |
| UGT3A2 | 1.534974 | 0.004969 |
| TAF4B | 1.534832 | 0.024253 |
| ZNF569 | 1.533556 | 0.018713 |
| TBX3 | 1.530234 | 0.026592 |
| CIRH1A | 1.527374 | 0.000892 |
| HAP1 | 1.523164 | 0.010972 |
| APPBP2 | 1.520219 | 0.005031 |
| LIPI | 1.516893 | 0.013224 |
| INO80 | 1.516602 | 0.012764 |
| AMPD1 | 1.516383 | 0.020486 |
| GMFB | 1.514557 | 0.018772 |
| GRAP | 1.511509 | 0.012945 |
| DGAT1 | 1.508633 | 0.017394 |
| MAGI3 | 1.508163 | 0.019868 |
| SPATA19 | 1.507931 | 0.037932 |
| SETBP1 | 1.50693 | 0.03283 |
| TPP2 | 1.502584 | 0.002668 |
| MED26 | 1.49758 | 0.000321 |
| HBE1 | 1.495728 | 0.014616 |
| ENAH | 1.495261 | 0.013483 |
| TP53BP1 | 1.494149 | 0.011918 |
| TSPY26P | 1.494099 | 0.000716 |
| SMURF2 | 1.489544 | 0.027369 |
| CHD7 | 1.487185 | 0.009174 |
| PTCRA | 1.48606 | 0.0197 |
| NFX1 | 1.482662 | 0.004666 |
| IZUMO4 | 1.482513 | 0.025878 |
| AKAP3 | 1.479818 | 0.004772 |
| IL17A | 1.4785 | 0.012147 |
| PPP1R13B | 1.475146 | 0.001458 |
| TLK2 | 1.468212 | 0.005055 |
| MIXL1 | 1.467589 | 0.007736 |
| DDR2 | 1.46695 | 0.037811 |
| SSBP2 | 1.466343 | 0.032329 |
| HS3ST3B1 | 1.466008 | 0.004335 |
| C7orf74 | 1.465862 | 0.039426 |
| SUV39H1 | 1.465115 | 0.020003 |
| TENC1 | 1.464056 | 0.041546 |
| RCAN2 | 1.463935 | 0.043492 |
| CTLA4 | 1.46292 | 0.034874 |
| LY6H | 1.462397 | 0.028571 |
| SRPK3 | 1.460491 | 0.032013 |
| FAM169B | 1.459813 | 0.015018 |
| MYCBP2 | 1.455795 | 0.017488 |
| BCOR | 1.45476 | 0.01168 |
| FZD4 | 1.448551 | 0.042553 |
| CHPF | 1.445919 | 0.044114 |
| CAMSAP2 | 1.441948 | 0.042961 |
| CUEDC1 | 1.439261 | 0.025797 |
| MEGF11 | 1.439017 | 0.02596 |
| SERPINA12 | 1.438263 | 0.006917 |
| ZPBP | 1.436809 | 0.003958 |
| SOCS2 | 1.433491 | 0.039903 |
| SRSF6 | 1.42498 | 0.045641 |
| RAI1 | 1.422856 | 0.008414 |
| TRIB2 | 1.421619 | 0.011224 |
| TRIM46 | 1.410461 | 0.015522 |
| THOC1 | 1.404133 | 0.023197 |
| RUNX2 | 1.403604 | 0.008535 |
| CNOT3 | 1.403077 | 0.010155 |
| SSTR4 | 1.401768 | 0.033889 |
| ODZ2 | 1.399982 | 0.00935 |
| DCAF10 | 1.398421 | 0.014803 |
| CD300LG | 1.393495 | 0.005961 |
| KLF9 | 1.393147 | 0.020422 |
| HERC4 | 1.392381 | 0.011003 |
| MRGPRD | 1.388237 | 0.041399 |
| OR4D2 | 1.386662 | 0.008923 |
| TRIM9 | 1.378958 | 0.025056 |
| TEX28 | 1.377952 | 0.015437 |
| INMT | 1.372924 | 0.024764 |
| CSPG5 | 1.370373 | 0.027222 |
| ARL5B | 1.366496 | 0.047349 |
| POLR2M | 1.363067 | 0.038193 |
| ZNF585A | 1.362711 | 0.002936 |
| FBXL22 | 1.362572 | 0.009742 |
| PRPF19 | 1.360145 | 0.030601 |
| GATA1 | 1.360074 | 0.000258 |
| KCNV2 | 1.358734 | 0.010769 |
| MEOX1 | 1.358655 | 0.045416 |
| GDF10 | 1.356194 | 0.012019 |
| GRK5 | 1.351162 | 0.019321 |
| VPS54 | 1.351098 | 0.001692 |
| ARRDC3 | 1.351064 | 0.049191 |
| ZZZ3 | 1.350765 | 0.025737 |
| DNAH10 | 1.350165 | 0.028647 |
| RBBP6 | 1.349816 | 0.026085 |
| PANX1 | 1.346453 | 0.035613 |
| C13orf27 | 1.346395 | 0.022346 |
| LDB2 | 1.345308 | 0.014133 |
| ARID4B | 1.344608 | 0.005337 |
| KLK14 | 1.341714 | 0.017141 |
| SIAH1 | 1.341481 | 0.020174 |
| KLHDC2 | 1.340924 | 0.009289 |
| DNM1 | 1.333817 | 0.023519 |
| LOC283665 | 1.329052 | 0.032161 |
| FSCN1 | 1.323373 | 0.049533 |
| FKSG43 | 1.3211 | 0.036649 |
| PHRF1 | 1.320077 | 0.047456 |
| DNAJA4 | 1.319701 | 0.038417 |
| USP42 | 1.319077 | 0.005361 |
| FGF5 | 1.318826 | 0.027431 |
| TCP10L2 | 1.318307 | 0.042886 |
| YPEL4 | 1.314299 | 0.037849 |
| SLC10A4 | 1.313872 | 0.025711 |
| LOC338651 | 1.312064 | 0.009862 |
| TAF5L | 1.310182 | 0.016909 |
| INTS8 | 1.309879 | 0.004204 |
| LOC100506066 | 1.304984 | 0.000493 |
| LOC149351 | 1.304609 | 0.028929 |
| UBTF | 1.30038 | 0.03166 |
| AXIN1 | 1.299864 | 0.015552 |
| LOC100506507 | 1.298213 | 0.035084 |
| PKD2L2 | 1.297626 | 0.008996 |
| E2F3 | 1.296934 | 0.014035 |
| PTBP2 | 1.289918 | 0.006357 |
| KDM6A | 1.28883 | 0.026246 |
| ABHD2 | 1.287018 | 0.042235 |
| PTPRT | 1.284643 | 0.020031 |
| FAM166B | 1.279829 | 0.019951 |
| C18orf25 | 1.275618 | 0.013235 |
| DCN | 1.272868 | 0.042191 |
| IFRD1 | 1.271677 | 0.008118 |
| PRRC2A | 1.271654 | 0.005273 |
| PIK3IP1 | 1.271447 | 0.029075 |
| SCAI | 1.271141 | 0.008064 |
| PRPF3 | 1.271096 | 0.008237 |
| CFC1 | 1.269155 | 0.006663 |
| SERPINA9 | 1.267738 | 0.023027 |
| HMX1 | 1.265574 | 0.031491 |
| BMP3 | 1.261907 | 0.00805 |
| SCN4B | 1.260238 | 0.036968 |
| WDR12 | 1.258991 | 0.005605 |
| ARID1A | 1.257466 | 0.037357 |
| KIF2A | 1.249678 | 0.004577 |
| RNF19A | 1.247944 | 0.008285 |
| DPEP1 | 1.247246 | 0.029363 |
| OR5T2 | 1.246196 | 0.041262 |
| FAM107A | 1.245359 | 0.015832 |
| CCNJ | 1.244502 | 0.016103 |
| LOC401176 | 1.243979 | 0.029871 |
| C17orf63 | 1.243057 | 0.004414 |
| HHATL | 1.238241 | 0.012247 |
| LOC440337 | 1.236854 | 0.038581 |
| HBBP1 | 1.236024 | 0.040529 |
| CSNK1G3 | 1.235186 | 0.037225 |
| STK35 | 1.23499 | 0.007501 |
| CNOT4 | 1.230484 | 0.0156 |
| RBBP5 | 1.229548 | 0.038846 |
| PCBP4 | 1.227293 | 0.02403 |
| LOC100129775 | 1.226337 | 0.015024 |
| LOC343052 | 1.226215 | 0.038923 |
| C7orf51 | 1.22504 | 0.014695 |
| TRPC2 | 1.224192 | 0.036719 |
| LOC100128922 | 1.223682 | 0.012791 |
| ZSWIM6 | 1.223474 | 0.048059 |
| PARP8 | 1.222217 | 0.01628 |
| SMAD2 | 1.221429 | 0.017532 |
| CHST10 | 1.220879 | 0.013758 |
| CHIC2 | 1.220244 | 0.035256 |
| LCE3C | 1.220162 | 0.01102 |
| B4GALNT1 | 1.219227 | 0.049017 |
| NDST4 | 1.219194 | 0.022028 |
| ROCK1 | 1.21915 | 0.040227 |
| OR4N4 | 1.217194 | 0.008545 |
| FZD5 | 1.216055 | 0.017352 |
| LOC100505978 | 1.213818 | 0.006597 |
| SEMA5B | 1.213713 | 0.010006 |
| MPO | 1.210222 | 0.010192 |
| FRMD8 | 1.209813 | 0.012259 |
| AFF4 | 1.208468 | 0.020288 |
| NLK | 1.208152 | 0.031002 |
| KIN | 1.204852 | 0.022084 |
| AKAP9 | 1.203481 | 0.035479 |
| CROCCP3 | 1.203377 | 0.036325 |
| PAFAH1B1 | 1.201838 | 0.004243 |
| NPFFR2 | 1.201131 | 0.014604 |
| DCP2 | 1.200994 | 0.033334 |
| SPTB | 1.200414 | 0.040356 |
| POU5F1P4 | 1.199869 | 0.020451 |
| ACAP3 | 1.199311 | 0.049745 |
| CD300E | 1.196511 | 0.005015 |
| IP6K1 | 1.195679 | 0.045377 |
| SLC25A23 | 1.193708 | 0.021612 |
| LOC100505813 | 1.189559 | 0.037603 |
| OSBPL7 | 1.188575 | 0.027378 |
| LOC100509498 | 1.187638 | 0.048582 |
| MAP2K4 | 1.184605 | 0.012765 |
| OR2T8 | 1.184192 | 0.049876 |
| SEMA4C | 1.181804 | 0.043186 |
| SKI | 1.175064 | 0.046865 |
| HNRNPUL2 | 1.173411 | 0.012075 |
| C11orf84 | 1.172494 | 0.010702 |
| NAA15 | 1.169595 | 0.031737 |
| YLPM1 | 1.16812 | 0.021619 |
| SHISA2 | 1.16789 | 0.023573 |
| SHB | 1.165427 | 0.023865 |
| DSG4 | 1.164064 | 0.016587 |
| IRX4 | 1.162828 | 0.005067 |
| HOXD9 | 1.162708 | 0.048421 |
| ISY1 | 1.161374 | 0.037346 |
| MASP2 | 1.161275 | 0.035422 |
| SGK1 | 1.160792 | 0.042419 |
| BCL6B | 1.158814 | 0.048112 |
| RNF157 | 1.156171 | 0.048964 |
| ANO2 | 1.156007 | 0.037733 |
| OR2M2 | 1.153764 | 0.012077 |
| HOXB1 | 1.152376 | 0.018725 |
| PIK3R1 | 1.151473 | 0.03555 |
| TAF1B | 1.150665 | 0.010399 |
| KIAA1370 | 1.149296 | 0.017174 |
| PPP5C | 1.149038 | 0.02505 |
| SEMA3F | 1.145515 | 0.037221 |
| LOC100131067 | 1.145007 | 0.013984 |
| C3orf20 | 1.144169 | 0.03353 |
| TTC5 | 1.14395 | 0.03759 |
| KLHDC1 | 1.143271 | 0.033279 |
| RASA1 | 1.142368 | 0.035581 |
| ARFGEF1 | 1.139689 | 0.004408 |
| SLC4A9 | 1.138116 | 0.031597 |
| DEFT1P | 1.135407 | 0.022844 |
| LINC00341 | 1.134502 | 0.029244 |
| ZNF592 | 1.132946 | 0.002321 |
| PDS5B | 1.132071 | 0.047396 |
| PCGF3 | 1.129035 | 0.007489 |
| RNF2 | 1.127584 | 0.014995 |
| MLL5 | 1.126546 | 0.020379 |
| ZMYM2 | 1.125283 | 0.005128 |
| FAM92A3 | 1.124906 | 0.00885 |
| PHF2 | 1.124096 | 0.017763 |
| MAPK8 | 1.123524 | 0.029463 |
| KRTAP4-8 | 1.122683 | 0.037017 |
| ZNF330 | 1.120329 | 0.017494 |
| CTDSPL2 | 1.118915 | 0.046529 |
| LOC284260 | 1.112526 | 0.017848 |
| KAT2B | 1.110896 | 0.048427 |
| PRRC2C | 1.110371 | 0.03976 |
| CDC73 | 1.109029 | 0.023629 |
| RNMT | 1.107747 | 0.041538 |
| MMD2 | 1.103322 | 0.017802 |
| IGIP | 1.10092 | 0.010783 |
| CECR5-AS1 | 1.100588 | 0.031182 |
| TGFB1 | 1.099636 | 0.033643 |
| CHRNG | 1.098124 | 0.006229 |
| CHRM2 | 1.097821 | 0.019413 |
| SMARCAD1 | 1.097506 | 0.011366 |
| NR2F6 | 1.097231 | 0.045546 |
| ITIH6 | 1.096994 | 0.038586 |
| LOC100128361 | 1.09697 | 0.032838 |
| TAF8 | 1.095362 | 0.022466 |
| DTNB | 1.093469 | 0.01633 |
| C2orf53 | 1.093029 | 0.02424 |
| OR11G2 | 1.091438 | 0.032947 |
| RLTPR | 1.091203 | 0.044306 |
| IWS1 | 1.090261 | 0.008911 |
| CWC15 | 1.08763 | 0.015887 |
| OTOS | 1.086925 | 0.020801 |
| KRTAP4-6 | 1.086301 | 0.038016 |
| C6orf154 | 1.085476 | 0.049927 |
| LEMD3 | 1.084644 | 0.037616 |
| API5 | 1.084242 | 0.047481 |
| SOHLH1 | 1.083876 | 0.015865 |
| SPO11 | 1.082997 | 0.018382 |
| TNRC6C | 1.08175 | 0.033077 |
| RNF111 | 1.081662 | 0.037056 |
| PPP2R2D | 1.081408 | 0.043789 |
| OSR1 | 1.080822 | 0.002723 |
| RBPMS | 1.080593 | 0.049343 |
| CCNT2 | 1.080585 | 0.040633 |
| PCDHGA9 | 1.078146 | 0.002385 |
| NIPBL | 1.077324 | 0.041425 |
| ARIH2 | 1.077018 | 0.014097 |
| C17orf74 | 1.076031 | 0.045104 |
| NLRC3 | 1.075152 | 0.049529 |
| PCGF1 | 1.074503 | 0.014876 |
| EPN2 | 1.073378 | 0.015463 |
| RSRC2 | 1.07 | 0.001688 |
| MLLT10 | 1.068991 | 0.048578 |
| SPTBN1 | 1.068465 | 0.048066 |
| SYN2 | 1.06751 | 0.024353 |
| POU6F2 | 1.065712 | 0.012175 |
| AGXT2L1 | 1.065077 | 0.024038 |
| BRD1 | 1.06426 | 0.001803 |
| AKIRIN2 | 1.063696 | 0.017317 |
| UBFD1 | 1.062477 | 0.036983 |
| NOLC1 | 1.06033 | 0.04446 |
| MOV10 | 1.06029 | 0.024382 |
| DEFA4 | 1.059261 | 0.049411 |
| GPR1 | 1.059128 | 0.028794 |
| LOC100509814 | 1.05866 | 0.007108 |
| SH2B1 | 1.058612 | 0.037916 |
| TDRG1 | 1.057815 | 0.008482 |
| C3orf51 | 1.056831 | 0.029723 |
| VPS36 | 1.055519 | 0.046234 |
| EPC2 | 1.054139 | 0.008352 |
| GUF1 | 1.052942 | 0.02751 |
| MYH7 | 1.052563 | 0.021508 |
| WIPF3 | 1.051936 | 0.017925 |
| DBC1 | 1.051368 | 0.015368 |
| GMPS | 1.051101 | 0.014759 |
| CSNK2A2 | 1.050928 | 0.046188 |
| BRMS1L | 1.050627 | 0.022499 |
| CNNM1 | 1.049166 | 0.041692 |
| FLJ40606 | 1.048115 | 0.028836 |
| AGFG1 | 1.047872 | 0.031154 |
| SNRNP48 | 1.046229 | 0.044869 |
| CLCN2 | 1.046172 | 0.025592 |
| RPS6KA3 | 1.044926 | 0.025889 |
| IFNA5 | 1.03924 | 0.043883 |
| KDM5B | 1.038724 | 0.035587 |
| MPP7 | 1.038528 | 0.01974 |
| ZC3H14 | 1.034461 | 0.035522 |
| MLL4 | 1.032333 | 0.046114 |
| C15orf38 | 1.029417 | 0.009731 |
| PPY2 | 1.028547 | 0.028922 |
| PRR12 | 1.028255 | 0.017415 |
| BRAF | 1.027562 | 0.01261 |
| BOC | 1.027446 | 0.046735 |
| SPSB1 | 1.026851 | 0.043976 |
| C19orf67 | 1.026254 | 0.030071 |
| SLC46A2 | 1.025652 | 0.035313 |
| UBL4A | 1.024896 | 0.03505 |
| CLK2 | 1.024698 | 0.03777 |
| DEFB129 | 1.024092 | 0.046118 |
| PMS2 | 1.023821 | 0.028386 |
| PCDHGC4 | 1.022905 | 0.01241 |
| XPOT | 1.020011 | 0.014287 |
| IPO7 | 1.019706 | 0.026532 |
| C19orf80 | 1.018277 | 0.010786 |
| SLC22A9 | 1.017976 | 0.040307 |
| WDR83 | 1.013535 | 0.022454 |
| CCDC39 | 1.011456 | 0.042789 |
| SENP5 | 1.011253 | 0.038817 |
| TCP10 | 1.01016 | 0.03088 |
| PPAN | 1.006579 | 0.035053 |
| CLPX | 1.005953 | 0.017709 |
| C13orf41 | 1.005391 | 0.023241 |
| C10orf25 | 1.003183 | 0.009284 |
| ENDOV | 1.003059 | 0.036426 |
| OR51Q1 | 1.001798 | 0.019358 |
| SYTL5 | 1.00162 | 0.02055 |
