# Supplementary material for: Immunity-Related Gene Signature Identifies Subtypes Benefitting From Adjuvant Chemotherapy or Potentially Responding to PD1/PD-L1 Blockage in Pancreatic Cancer
Source: Front Cell Dev Biol. 2021 Jun 23;9:682261. doi: 10.3389/fcell.2021.682261 (PMC8264789; doi:10.3389/fcell.2021.682261)
Supplement: Supplementary Table 5 — The gene name of each row from the heatmap in Figure 7B. [file Table_5.docx]

Supplementary Table 5. The gene name of each row from the heatmap in figure 7B

| Row | Gene name |
| --- | --- |
| Row1 | ABCA9 |
| Row2 | ABCG2 |
| Row3 | ACAP1 |
| Row4 | ACAP3 |
| Row5 | AKAP3 |
| Row6 | AKAP9 |
| Row7 | AMPD1 |
| Row8 | ANO2 |
| Row9 | APPBP2 |
| Row10 | ARID1A |
| Row11 | ARIH2 |
| Row12 | ARNTL |
| Row13 | ATP1A2 |
| Row14 | B4GALNT1 |
| Row15 | BACH2 |
| Row16 | BCL6B |
| Row17 | BCOR |
| Row18 | BMP3 |
| Row19 | BOC |
| Row20 | BPHL |
| Row21 | BRAF |
| Row22 | BRD1 |
| Row23 | BRMS1L |
| Row24 | C10orf25 |
| Row25 | C18orf25 |
| Row26 | C3orf20 |
| Row27 | CCDC39 |
| Row28 | CD300LG |
| Row29 | CDK17 |
| Row30 | CERS4 |
| Row31 | CFC1 |
| Row32 | CHD5 |
| Row33 | CHD7 |
| Row34 | CHRM2 |
| Row35 | CHST10 |
| Row36 | CNOT4 |
| Row37 | CROCCP3 |
| Row38 | CRYAB |
| Row39 | CSPG5 |
| Row40 | CTLA4 |
| Row41 | DNM1 |
| Row42 | DPYSL5 |
| Row43 | DTNA |
| Row44 | ELMOD1 |
| Row45 | ENDOV |
| Row46 | EPC2 |
| Row47 | EPHA10 |
| Row48 | F7 |
| Row49 | FAM107A |
| Row50 | FAM166B |
| Row51 | FAM184A |
| Row52 | FBLN7 |
| Row53 | FBXL22 |
| Row54 | FGF10 |
| Row55 | FZD4 |
| Row56 | GATA1 |
| Row57 | GFRA1 |
| Row58 | GJC1 |
| Row59 | GNAZ |
| Row60 | GPM6A |
| Row61 | GPR18 |
| Row62 | GPR88 |
| Row63 | GRAP |
| Row64 | GRK5 |
| Row65 | HAP1 |
| Row66 | HAS1 |
| Row67 | HHATL |
| Row68 | HOXD9 |
| Row69 | HS3ST3B1 |
| Row70 | HTR5A |
| Row71 | IGIP |
| Row72 | INMT |
| Row73 | IP6K1 |
| Row74 | IRF2BP1 |
| Row75 | ITIH6 |
| Row76 | IZUMO4 |
| Row77 | JAM2 |
| Row78 | KAT2B |
| Row79 | KCNC1 |
| Row80 | KCNH2 |
| Row81 | KCNJ8 |
| Row82 | KLF12 |
| Row83 | KLF9 |
| Row84 | KLHDC1 |
| Row85 | KLHDC2 |
| Row86 | KLHL29 |
| Row87 | LDB2 |
| Row88 | LILRA4 |
| Row89 | LIPI |
| Row90 | LMO1 |
| Row91 | LRFN5 |
| Row92 | LY6H |
| Row93 | MAP2K4 |
| Row94 | MARK1 |
| Row95 | MASP2 |
| Row96 | MED26 |
| Row97 | MEOX1 |
| Row98 | MEOX2 |
| Row99 | MEX3B |
| Row100 | MLLT10 |
| Row101 | MLLT3 |
| Row102 | MMD2 |
| Row103 | MPDZ |
| Row104 | MRGPRD |
| Row105 | MYCBP2 |
| Row106 | NAV3 |
| Row107 | NDST4 |
| Row108 | NEFH |
| Row109 | NLK |
| Row110 | NLRC3 |
| Row111 | NPFFR2 |
| Row112 | NRN1 |
| Row113 | OR2T8 |
| Row114 | OSR1 |
| Row115 | PACSIN1 |
| Row116 | PAFAH1B1 |
| Row117 | PCDHGC4 |
| Row118 | PDS5B |
| Row119 | PGLYRP1 |
| Row120 | PGLYRP2 |
| Row121 | PHF2 |
| Row122 | PHYHIPL |
| Row123 | PIK3R1 |
| Row124 | PKD2L2 |
| Row125 | PKNOX2 |
| Row126 | PMS2 |
| Row127 | POU6F2 |
| Row128 | PPAN |
| Row129 | PRKCQ |
| Row130 | PTBP2 |
| Row131 | PTPRT |
| Row132 | RAI1 |
| Row133 | RBBP6 |
| Row134 | RCAN2 |
| Row135 | REV3L |
| Row136 | RNF157 |
| Row137 | ROBO4 |
| Row138 | SAMD3 |
| Row139 | SCAI |
| Row140 | SCN4B |
| Row141 | SEMA5B |
| Row142 | SEMA6A |
| Row143 | SEMA6D |
| Row144 | SERPINA12 |
| Row145 | SETBP1 |
| Row146 | SGCD |
| Row147 | SH2B1 |
| Row148 | SHANK3 |
| Row149 | SIX6 |
| Row150 | SLC10A4 |
| Row151 | SLC22A9 |
| Row152 | SMAD2 |
| Row153 | SMARCAD1 |
| Row154 | SMTN |
| Row155 | SNRNP48 |
| Row156 | SOBP |
| Row157 | SOCS2 |
| Row158 | SORBS1 |
| Row159 | SOX5 |
| Row160 | SPTB |
| Row161 | SRGAP3 |
| Row162 | SRSF6 |
| Row163 | SSBP2 |
| Row164 | SSTR4 |
| Row165 | STIM2 |
| Row166 | SYN2 |
| Row167 | TAC1 |
| Row168 | TBX21 |
| Row169 | TBX3 |
| Row170 | TCP10 |
| Row171 | TCP10L2 |
| Row172 | THOC1 |
| Row173 | TIE1 |
| Row174 | TLE6 |
| Row175 | TLK2 |
| Row176 | TNRC6C |
| Row177 | TP53BP1 |
| Row178 | TRIM46 |
| Row179 | TRIM9 |
| Row180 | TSPY26P |
| Row181 | TTC5 |
| Row182 | UBXN11 |
| Row183 | UNC45B |
| Row184 | USP42 |
| Row185 | VTN |
| Row186 | WDR83 |
| Row187 | WIPF3 |
| Row188 | YLPM1 |
| Row189 | YPEL4 |
| Row190 | ZBTB10 |
| Row191 | ZBTB16 |
| Row192 | ZC3H14 |
| Row193 | ZNF280D |
| Row194 | ZNF569 |
| Row195 | ZNF585A |
| Row196 | ZNF625 |
| Row197 | ZPBP |
| Row198 | ZZZ3 |
